# Metal Complexes of a 5-Nitro-8-Hydroxyquinoline-Proline Hybrid with Enhanced Water Solubility Targeting Multidrug Resistant Cancer Cells

**DOI:** 10.3390/ijms24010593

**Published:** 2022-12-29

**Authors:** Tamás Pivarcsik, Vivien Pósa, Hilda Kovács, Nóra V. May, Gabriella Spengler, Szonja P. Pósa, Szilárd Tóth, Zeinab Nezafat Yazdi, Csilla Özvegy-Laczka, Imre Ugrai, István Szatmári, Gergely Szakács, Éva A. Enyedy

**Affiliations:** 1 MTA-SZTE Lendület Functional Metal Complexes Research Group, University of Szeged, Dóm tér 7, H-6720 Szeged, Hungary; 2 Department of Inorganic and Analytical Chemistry, Interdisciplinary Excellence Centre, University of Szeged, Dóm tér 7, H-6720 Szeged, Hungary; 3 Centre for Structural Science, Research Centre for Natural Sciences, Eötvös Loránd Research Network, Magyar Tudósok krt. 2, H-1117 Budapest, Hungary; 4 Department of Medical Microbiology, Albert Szent-Györgyi Health Center, Albert Szent-Györgyi Medical School, University of Szeged, Semmelweis u. 6, H-6725 Szeged, Hungary; 5 Drug Resistance Research Group, Institute of Enzymology, Research Centre for Natural Sciences, Eötvös Loránd Research Network, Magyar Tudósok krt. 2, H-1117 Budapest, Hungary; 6 National Laboratory for Drug Research and Development, Magyar Tudósok krt. 2, H-1117 Budapest, Hungary; 7 Institute of Pharmaceutical Chemistry and Stereochemistry Research Group, Eötvös Loránd Research Network, University of Szeged, Eötvös u. 6, H-6720 Szeged, Hungary; 8 Institute of Cancer Research, Medical University of Vienna, Borschkegasse 8a, A-1090 Vienna, Austria

**Keywords:** speciation, solution structure, organometallic complexes, cytotoxicity, multidrug resistance, 8-hydroxyquinoline

## Abstract

Multidrug resistance (MDR) in cancer is one of the major obstacles of chemotherapy. We have recently identified a series of 8-hydroxyquinoline Mannich base derivatives with MDR-selective toxicity, however with limited solubility. In this work, a novel 5-nitro-8-hydroxyquinoline-proline hybrid and its Rh(η^5^-C_5_Me_5_) and Ru(η^6^-*p*-cymene) complexes with excellent aqueous solubility were developed, characterized, and tested against sensitive and MDR cells. Complex formation of the ligand with essential metal ions was also investigated using UV-visible, circular dichroism, ^1^H NMR (Zn(II)), and electron paramagnetic resonance (Cu(II)) spectroscopic methods. Formation of mono and bis complexes was found in all cases with versatile coordination modes, while tris complexes were also formed with Fe(II) and Fe(III) ions, revealing the metal binding affinity of the ligand at pH 7.4: Cu(II) > Zn(II) > Fe(II) > Fe(III). The ligand and its Rh(III) complex displayed enhanced cytotoxicity against the resistant MES-SA/Dx5 and Colo320 human cancer cell lines compared to their chemosensitive counterparts. Both organometallic complexes possess high stability in solution, however the Ru(II) complex has lower chloride ion affinity and slower ligand exchange processes, along with the readiness to lose the arene ring that is likely connected to its inactivity.

## 1. Introduction

Chemotherapy is still a common and effective way to treat many types of cancer, despite limitations including serious side-effects and resistance. Multidrug resistance (MDR) of cancer cells against structurally different anticancer agents is a major problem often leading to unsuccessful chemotherapy [1]. MDR is often linked to the overexpression of ATP-binding cassette (ABC) transporters mediating the efflux of chemotherapeutics from cancer cells [1,2,3]. P-glycoprotein (ABCB1/P-gp) is a widely investigated and characterized ABC transporter, whose inhibition was generally believed to represent an effective treatment strategy. Effective inhibition of P-gp function by four generations of agents has been widely described. Unfortunately, despite the enormous efforts, many drug candidates have failed during clinical trials [4]. A recently proposed strategy to overcome multidrug resistance is to target the collateral sensitivity of otherwise resistant cells by MDR-selective compounds. In earlier work, we designed a library containing a high number (>500) of 8-hydroxyquinoline (HQ) derivatives possessing variable MDR-selective toxicity [5,6,7]. The toxicity of MDR-selective 8-HQ derivatives is increased, rather than decreased, by P-gp [8].

The advantageous medical aspects of HQ-s are not new, as HQ derivatives are reported to have a wide-range of bioactivities including anticancer [9], antibacterial [10] or antiviral [11] properties. However, many HQ derivatives suffer from poor water solubility which is an unfavourable pharmaceutical feature. Previously, we have reported 8-hydroxyquinoline derived Mannich bases possessing excellent water solubility related to their zwitterionic form [12,13]. Additionally, the CH_2_-N subunit at the seventh position on the HQ ring proved to be beneficial in terms of MDR selectivity [5,7]. We have shown that the cytotoxicity and MDR-selectivity of 8-hydroxyquinoline Mannich bases are related to their complex formation with essential metal ions [5,7,14]. We have characterized the acid–base properties and metal binding abilities of a series of HQs derived Mannich bases with tertiary amines [5,7,14]. Among others, a relationship was found between the p*K*_a_ values (mainly for the OH group) and the cytotoxicity against MDR cells in the case of a library consisting of 120 derivatives [7]. Our studies underlined the importance of complex formation with iron and copper ions in MDR selective toxicity via iron deprivation and/or the formation of redox-active copper complexes [5,7,15]. Besides iron and copper, zinc is also essential for the growth and proliferation of the rapidly dividing cancer cells, and the HQ-based clioquinol was demonstrated to target zinc to lysosomes in cancer cells and to act as a zinc ionophore [16]; however, the 5-nitro-8-hydroxyquinoline (nitroxoline) lacks the latter activity [17].

HQs are also strong chelators for other metal ions, and so far, countless anticancer complexes of transition metal ions have been developed and investigated [12,13,18,19,20,21,22,23,24]. Complexation can alter various physico-chemical properties (e.g., size, charge, lipophilicity, solubility, stability, protein binding); therefore, the pharmacokinetics of a drug, and consequently modified pharmacodynamics (different targets and mechanisms of action) are likely. The strong coordination bond between the metal ion and the ligand can be crucial regarding the biological activity of the complexes, since high stability can promote the transportation of the ligand to the target biomolecule, or the whole complex can act as a substrate or inhibitor. The high stability of half-sandwich Ru(η^6^-*p*-cymene) (RuCym) and Rh(η^5^-C_5_Me_5_) (RhCp*) complexes with HQs in solution is a characteristic feature [12,13,23], and these types of complexes are widely investigated due to their potential in cancer treatment [12,13,22,23,24,25,26,27]. These piano–stool complexes contain the bidentately coordinated HQ type ligand and a chlorido co-ligand. The systematic structural variation of the components can lead to diverse physico-chemical, and therefore biological properties. The beneficial effect of a halogen substituent, mainly at the fifth position of the HQ scaffold, was confirmed previously [5,6]. The nitro group has similar properties, such as in the case of nitroxoline which is widely studied as an antibacterial and anticancer agent [17,28]. However, the solubilities of the halogen and nitro HQ derivatives in water are also limited. 

In our previous reports, we developed and characterized novel 8-hydroxyquinoline Mannich bases with a chlorine substituent possessing excellent water solubility, in addition to RhCp* and RuCym complexes [12,13]. The RhCp* complexes displayed potent cytotoxicity in Colo205 and Colo320 human adeno carcinoma cell lines with a selectivity ratio ~2 over against the non-cancerous MRC-5 cell line, while lower activity could be observed in the case of RuCym complexes compared to the free ligand. Our studies suggested the possibility of the loss of the *p*-cymene ligand that might contribute to the ineffectiveness of the latter complex.

**Scheme 1:**
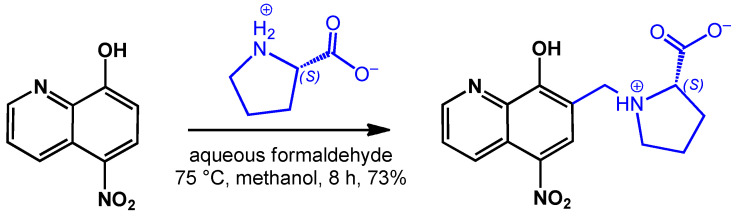
Synthetic route for the preparation of HQNO_2_-L-Pro.

Herein, we report the development of a novel 8-hydroxyquinoline Mannich base ((2S)-1-((8-hydroxy-5-nitroquinolin-7-yl)methyl)pyrrolidin-1-ium-2-carboxylate (HQNO_2_-L-Pro) (Scheme 1) with a nitro substituent having excellent aqueous solubility in addition to its RhCp* and RuCym complexes. Their anticancer properties on chemo-naive and multidrug resistant cell lines were investigated along with a detailed solution equilibrium study. As HQNO_2_-L-Pro is anionic at physiological pH, its inhibitory effect on the function of organic anion transporting polypeptides (OATPs) was also monitored. The complex formation of HQNO_2_-L-Pro with essential metal ions, namely Cu(II), Fe(III), Fe(II), and Zn(II), was characterized to gain insight into the relationship between the metal-binding ability of 8-hydroxyquinoline Mannich base targeting therapy resistant cancer, and MDR selective toxicity.

## 2. Results and Discussion

### 2.1. Synthesis and Characterization of the HQNO_2_-L-Pro ligand and Its Ru(η^6^-p-cymene) and Rh(η^5^-C_5_Me_5_) Complexes

The incorporation of the zwitterionic amino acids, such as proline or homoproline, in the ligand scaffold of 5-chloro-8-hydroxyquinoline was reported to efficiently increase aqueous solubility in our previous papers [12,13]. The exchange of the chlorine substituent to a nitro group can further modify solubility and lipophilicity, and it can also alter the mechanism of action due to its redox properties, since nitro compounds are often activated via a bioreductive process [29]. Therefore, HQNO_2_-L-Pro was synthesized by aminoalkylation of 5-nitro-8-hydroxyquinoline with L-proline in the presence of aqueous formaldehyde (Scheme 1). This latter modified Mannich reaction was carried out in methanol, while for the transformation of the starting compounds 8 h was needed at reflux temperature. The formed HQNO_2_-L-Pro was isolated from the solution by re-crystallization. The structure and purity of the compound were confirmed by ^1^H and ^13^C NMR spectroscopy and electrospray mass spectrometry (ESI-MS) measurements (see Materials and Methods section and NMR spectra in Figures S1 and S2).

**Scheme 2:**
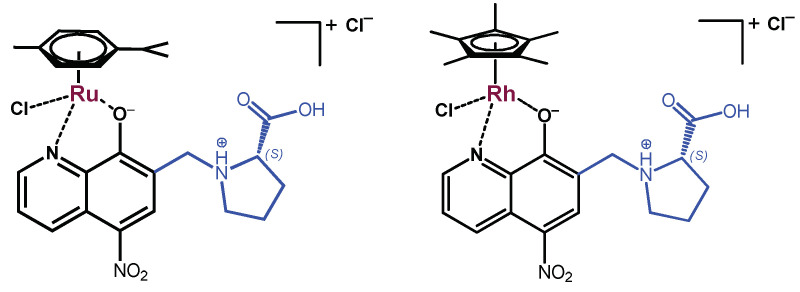
Chemical formulae of the isolated complexes: [RuCym(HQNO_2_-L-Pro)Cl]Cl and [RhCp*(HQNO_2_-L-Pro)Cl]Cl.

The organometallic Ru(η^6^-*p*-cymene) (RuCym) and Rh(η^5^-C_5_Me_5_) (RhCp*) complexes of HQNO_2_-L-Pro (Scheme 2) were obtained by mixing the ligand with a half-equivalent of the corresponding dimeric precursor [metal(arene)Cl_2_]_2_ in water (RhCp*, pH_0_ ~3) or in nitric acid (RuCym, pH_0_ ~2). After 24 h reaction time, the solvent was evaporated, then the obtained solid was dissolved in dichloromethane (RuCym complex) or in methanol (RhCp* complex). Precipitation was carried out with *n*-hexane or diethyl ether. The formed metal complexes were filtered out and dried. The structure and purity of the complexes were characterized by ^1^H and ^13^C NMR spectroscopy in D_2_O or DMSO-*d*_6_ (Figures S3–S6). Quantitative-NMR (via the use of maltol as internal standard, Figure S7) and ESI-MS methods were applied to reveal their stoichiometry. Additionally, capillary zone electrophoresis (CZE) was also applied to check the purity of the complexes (Figure S8). The electropherograms, quantitative-NMR, and ESI-MS spectra indicate the formation of the complexes with composition [RuCym(HQNO_2_-L-Pro)Cl]Cl and [RhCp*(HQNO_2_-L-Pro)Cl]Cl (Scheme 2). Similar complexes were isolated in the case of the HQCl-D-(h)Pro analogues [13], where bidentate coordination via the quinoline nitrogen and hydroxylate oxygen was proven by X-ray crystallography.

### 2.2. Solution Characterization of the HQNO_2_-L-Pro

**Figure 1:**
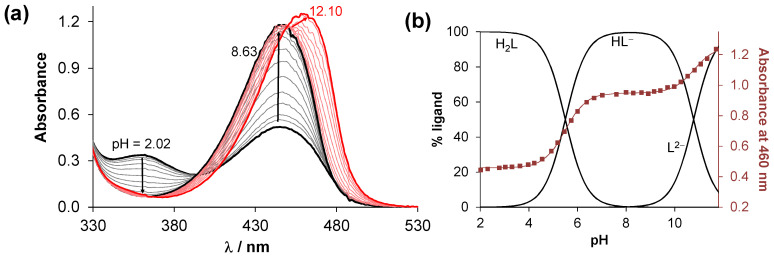
(**a**) UV-vis spectra of HQNO_2_-L-Pro recorded in the pH range 2–12. (**b**) Concentration distribution curves calculated with the determined p*K*_a_ values and absorbance values at 460 nm (■) with the fitted line (solid line). {*c*_ligand_ = 40 μM; *I* = 0.20 M KNO_3_; *l* = 1 cm; *t* = 25.0 °C}.

Characterization of the protonation/deprotonation equilibrium processes of a bioactive molecule is important to reveal its actual chemical form and charge at a given pH. The proton dissociation constant (*K*_a_) is a key physico-chemical parameter since it has an impact on many biopharmaceutical characteristics such as lipophilicity, water solubility, and albumin binding, which in turn directly influences pharmacokinetics. The p*K*_a_ values of the protonated HQNO_2_-L-Pro were determined by pH-potentiometric, UV-vis (Figure 1a), and ^1^H NMR spectroscopic titrations (Table 1).

Although, the fully protonated form of this compound possesses four functional groups which may dissociate, namely COOH, quinolinium-NH^+^, OH, and the proline-NH^+^ (N_Pro_H^+^), only two p*K*_a_ values could be determined in the studied pH-range (2–12). Deprotonation of the COOH and quinolinium-NH^+^ groups most likely takes place at very acidic pH values, as reported for the analogous 5-chloro derivative [12,13]; thus, the determined p*K*_a_ constants are assigned to the OH and N_Pro_H^+^ moieties. The ^1^H NMR spectra, recorded at various pH values (Figure S9a), show that the peaks of the quinoline ring protons are upload shifted in the pH range 4.5–7.0 but were unchanged at higher pH values, while the proline ring protons (Figure S9c) show significant changes only at pH > 10. Therefore, the lower p*K*_a_ belongs to the OH group, and the higher one to the proline-NH^+^.

It should be noted that HQNO_2_-L-Pro has considerably lower p*K*_a_ values in comparison to HQCl-L-Pro (p*K*_a_ (OH) = 7.76; p*K*_a_ (N_Pro_H^+^) > 11.5 [12]) due to the stronger electron-withdrawing ability of the nitro group over the chlorine, since a not merely inductive but resonance effect on the electronic system is also realized, especially in the para-location. As a result, HL^−^ is the sole species in the case of HQNO_2_-L-Pro at pH 7.4 (Figure 1b), while for HQCl-L-Pro 63.5% neutral and 36.5% negatively charged forms were found. Both the neutral and negatively charged forms of these compounds consist of the zwitterionic proline residue (COO^−^, N_Pro_H^+^) resulting in excellent solubility (*S*) in water at physiological pH (*S*_7.4_ > 10 mM). It should be also noted that an intramolecular hydrogen bond is assumed to form between the deprotonated O^−^ moiety and the protonated proline nitrogen in the case of the HL^−^ species, which also contributes to the relatively low p*K*_a_ (OH) and high p*K*_a_ (N_Pro_H^+^) values.

**Figure 2:**
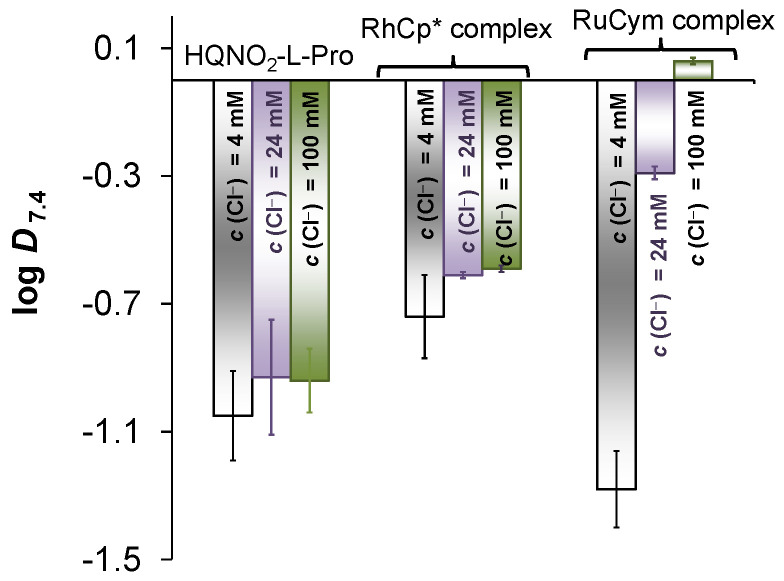
*n*-Octanol/water distribution coefficients at pH = 7.4 (log *D*_7.4_) determined for HQNO_2_-L-Pro and its RhCp* and RuCym complexes at different chloride ion concentrations: 4 mM, 24 mM and 100 mM (indicated on the columns), which correspond to the chloride content of the blood serum, cytosol, and nucleus. {pH = 7.40 (20 mM phosphate buffer); *t* = 25.0 °C}.

The lipophilicity of HQNO_2_-L-Pro expressed as distribution coefficient (*D*) was determined at pH 7.4 (Figure 2, Table S1) and at pH 5.5 via the conventional *n*-octanol/water shake-flask method. The actual chloride ion concentration as well as the decreased pH have only a minor influence on the *D* values. However, at pH 5.5, which corresponds to the more acidic pH of the tumor microenvironment, 53% HL^−^ and 47% H_2_L are present; the compound displays similar lipophilicity as at pH 7.4. The neutral H_2_L form is also fairly hydrophilic due to COO^−^ and quinolinium-NH+ moieties. It is noteworthy that HQNO_2_-L-Pro was found to be undoubtedly more hydrophilic than HQCl-L-Pro (Table S1).

### 2.3. Complex Formation of the HQNO_2_-L-Pro Ligand with Essential Metal Ions

Since the anticancer activity of the title compound may be related to the in situ formation of metal complexes in biofluids, as it was found for related 8-hydroxyquinoline Mannich bases [5,14], complex formation equilibria of HQNO_2_-L-Pro in aqueous solution with Fe(III), Fe(II), Cu(II), and Zn(II) were investigated by UV-vis, circular dichroism (CD), EPR (for Cu(II)), ^1^H NMR (for Zn(II)) spectroscopic titrations, and the redox properties of the forming redox active iron and copper complexes were characterized by cyclic voltammetry.

#### 2.3.1. Solution Equilibrium of the Fe(III) and Fe(II) Complexes

UV-vis titrations were performed to determine the formation (overall stability) constants (*β*) for the complexes of HQNO_2_-L-Pro formed with Fe(III) and Fe(II) ions (Figures S10 and S11). In the latter case, titrations were performed in a laboratory glove box. The calculations were complicated by the overlapping feature of the ligand bands and the charge transfer bands of the complexes. Based on the absorption spectra Figures S10a and S11a), formation of mono, bis, and tris complexes were found in both chemical systems; obtained constants are collected in Table 2. In the complexes [M(HL)], [M(HL)_2_], [M(HL)_3_] coordination via the (N,O^−^) donor set is suggested, and the proton is attributed to the non-coordinating protonated proline nitrogen, while [M(L)] is formed by its deprotonation or is a mixed-hydroxido species. Based on the concentration distribution diagrams (see Figure S10b for Fe(III) and Figure S11b for Fe(II)), it can be concluded that the tris complexes are the dominant species at pH 7.4 in the case of Fe(III), while with Fe(II) both tris and bis-ligand species are formed under the conditions of the titrations.

**Figure 3:**
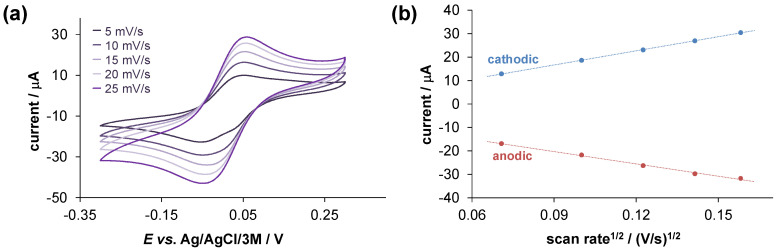
(**a**) Cyclic voltammograms of the Fe(III)–HQNO_2_-L-Pro system at various scan rates (indicated in the figure). (**b**) Current is plotted against the root square of the applied scan rate for the same system. Both curves are linear with similar slopes that suggest a diffusion-controlled electrode reaction in both oxidation states. {*c*_ligand_ = 1.5 mM, *c*_Fe(III)_ = 0.5 mM; pH = 7.40 (10 mM HEPES), *t* = 25 °C, *I* = 0.1 M (TBAN)}.

Cyclic voltametric measurements were performed to characterize the redox properties of the iron complexes. The voltammograms (Figure 3) were recorded at pH 7.4, at 1:3 metal-to-ligand ratio, and at much higher concentrations than those applied for UV-vis measurements (*c*_iron_ = 0.5 mM). Under this condition, 97% of Fe(III) is bound in the [Fe(HL)_3_] complex, and it revealed that in the case of Fe(II), the tris complex [Fe(HL)_3_]^−^ is the dominant (70%). The voltammograms displayed reversible processes (Table S2). The formal potential calculated for the Fe(III)/Fe(II) redox pair (*E*’_1/2_ = +215 ± 5 mV vs. NHE) is significantly more positive than that of the reference compound 8-hydroxyquinoline’s complexes (*E*’_1/2_ = −323 mV vs. NHE) [15]. Similarly, a more positive redox potential was found for the complexes of another Mannich base derivate (piperidine (7-(piperidin-1-ylmethyl)quinolin-8-ol) possessing MDR selective activity (*E*’_1/2_ = +21 mV vs. NHE [14]). The higher redox potential indicates a higher preference of HQNO_2_-L-Pro for Fe(II) in comparison to 8-hydroxyquinoline, which might have a role in the differences in their MDR selectivity. It should be also noted that no standalone redox processes were detected for HQNO_2_-L-Pro in the monitored potential range (−0.5–+0.5 V).

#### 2.3.2. Solution Equilibrium of the Cu(II) and Zn(II) Complexes

The reference compound 8-hydroxyquinoline forms mono and bis complexes with Cu(II) and Zn(II) coordinating via the (N,O^−^) donor set, which is well-documented in the literature [14,30,31]. Our aim was to study whether the proline and the nitro moieties in HQNO_2_-L-Pro have an impact on the speciation model and the stability of the complexes in solution.

**Figure 4:**
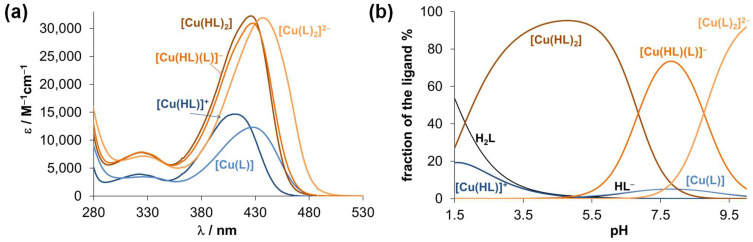
(**a**) Calculated molar UV-vis absorption spectra of the Cu(II) complexes with HQNO_2_-L-Pro. (**b**) Concentration distribution curves computed for the Cu(II)-HQNO_2_-L-Pro (1:2) system. H_2_L is the neutral form of the ligand (COO^−^, N_Pro_H^+^, N_quinoline_, OH). {*c*_ligand_ = 40 μM; *I* = 0.10 M KCl; *t* = 25.0 °C}.

UV-vis spectra were recorded at different Cu(II)-to-ligand ratios (Figure S12), revealing a more complicated speciation model than with 8-hydroxyquinoline. The evaluation of the spectral changes in the 280–530 nm wavelength range at pH < 10 suggested the formation of mono-ligand species [Cu(HL)]^+^ and [Cu(L)] in addition to bis-ligand complexes such as [Cu(LH)_2_], [Cu(LH)(L)]^−^ and [Cu(L)_2_]^2−^. Their formation constants are found in Table 2 and the computed molar absorbance spectra of the various species are shown in Figure 4a. In the partially protonated complexes ([Cu(HL)]^+^, [Cu(LH)_2_], [Cu(LH)(L)]^−^) the coordinated ligand most probably contains the protonated proline nitrogen, while in [Cu(L)] and [Cu(L)_2_]^2−^ the ligand is fully deprotonated.

Interestingly, the transformation of [Cu(HL)]^+^ → [Cu(L)] is accompanied by a significant red shift, and the latter species becomes dominant between pH 6.5 and 9.3 in samples with Cu:ligand = 1:1 ratio (see the constant absorbance values with λ_max_ 426 nm in this pH range in Figure S12c). On the other hand, the molar spectra of [Cu(HL)_2_] and [Cu(HL)(L)]^−^ are rather similar to each other and formation of [Cu(L)_2_]^2−^ results in an increase in the λ_max_ value. Notably, further spectral changes were observed at pH > 10 most probably due to the formation of mixed-hydroxido species. It was found that the λ_max_ values of the species depend on the actual protonation state of the coordinated ligand, actually more strongly than expected on the basis of the simple deprotonation of the non-chromophoric proline moiety. Therefore, additional measurements were performed focusing on the wavelength range of the d-d transition bands by UV-vis and CD spectroscopic titrations, and EPR spectroscopy was also applied to get insight into the coordination environment around the Cu(II) ion. Since these methods require higher total concentrations, we used a 30% (*v*/*v*) DMSO/H_2_O solvent mixture for the samples to ensure better solubility.

The CD spectra (Figure S13a,b) showed a weak negative band (λ_max_ ~704 nm; lowest value: −5 mdeg) in the pH range of the formation of [Cu(HL)]^+^ and [Cu(LH)_2_], and the negative peak becomes much stronger in the equimolar solution (−24 mdeg) with increasing pH as [Cu(L)] is formed. This finding suggests that the chiral proline moiety is involved in the coordination of these species (at least partly) under the applied conditions.

**Figure 5:**
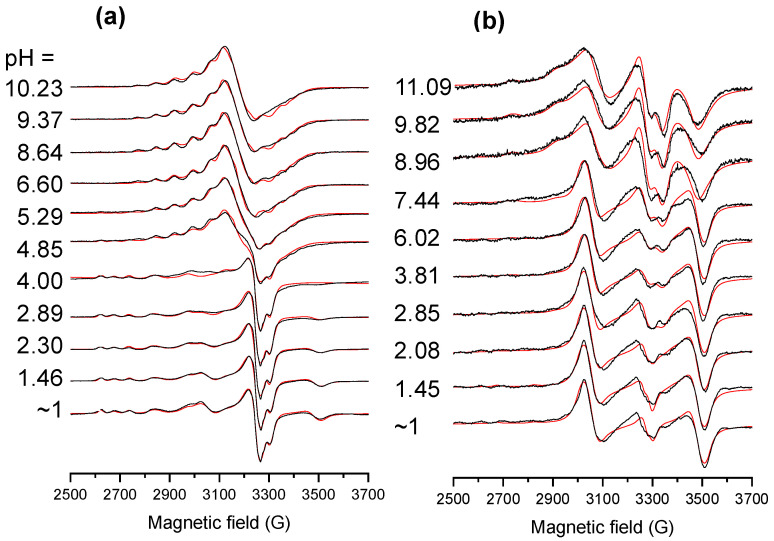
Experimental (black) and simulated (red) frozen solution EPR spectra recorded at various pH values for the Cu(II)-HQNO_2_-L-Pro system at (**a**) 1:1 and (**b**) (1:2) Cu(II):ligand ratio in 30% (*v*/*v*) DMSO/H_2_O. {*c*_ligand_ = 500 μM; *c*_Cu(II)_ = 500 or 250 μM; *I* = 0.10 M KCl; *T* = 77 K}.

Based on the simulation of the frozen EPR spectra (Figure 5), anisotropic EPR parameters (Table S3) and components spectra (Figure S14) were obtained. The species were identified as monomer I and II whose EPR parameters correspond well to mono- and bis-ligand complexes with (N,O^−^) coordination mode [14], respectively; however, three dinuclear species (I, II, III) were also found. The distribution of the various species based on the EPR data is shown in Figure S14. The dinuclear species I is formed at pH < ~4.5 at 1:1 ratio, practically together with monomer I identified as [CuLH]^+^ complex, and at pH < ~8 at 1:2 ratio. The dinuclear species II appears only at a 1:2 ratio at pH > 8, while species III is present at a 1:1 ratio at pH > 4. Based on the dipolar coupling (*D* values in Table S3), the distance of the Cu(II) ions (*d*_Cu-Cu_) in the dinuclear species could be calculated, and 4.1, 4.3, and 6.5 Å were obtained for I, II, and III, respectively. In species III, the equatorial planes of the Cu(II) ions are not parallel, and *g*-tenzor has a strong rhombic feature. Most probably [CuL] is present in the dimeric form [(CuL)_2_)] as species III, in which the deprotonated proline side chain binds to the neighbouring Cu(II) and vice versa, that also led to the stronger CD signal (Figure S13a). In the dinuclear species I, the equatorial planes of the metal ions are parallel, and its formation via the dimerization of the bis–ligand complex [Cu(LH)_2_] is feasible. Most probably, the dimerization of [Cu(LH)(L)]^−^ at 1:2 ratio also results in a dinuclear species with practically identical EPR parameters of a dimer of [Cu(LH)_2_]. Meanwhile, parallel to the formation of [Cu(L)_2_]^2−^, the dinuclear species II is present in the solution with a weaker optical activity. It should be noted that the monomeric and dinuclear species are in equilibrium in the whole pH range at 1:2 ratio (and at pH < ~4.5 at 1:1 ratio).

In all, the solution speciation of the Cu(II) complexes of HQNO_2_-L-Pro is rather different and more complicated in comparison to 8-hydroxyquinoline, since the proline substituent can be involved in the coordination as a bridging ligand forming dinuclear complexes. However, we have no proofs that these dinuclear species also exist in the more diluted solutions applied for the UV-vis titrations at 40 μM concentration, or under physiologically more relevant conditions.

The redox properties of the Cu(II) complex were monitored by CV measurements (Figure S15) and the obtained electrochemical data indicate irreversible processes; thus, the direct comparison to other Cu(II) complexes is not adequate.

**Figure 6:**
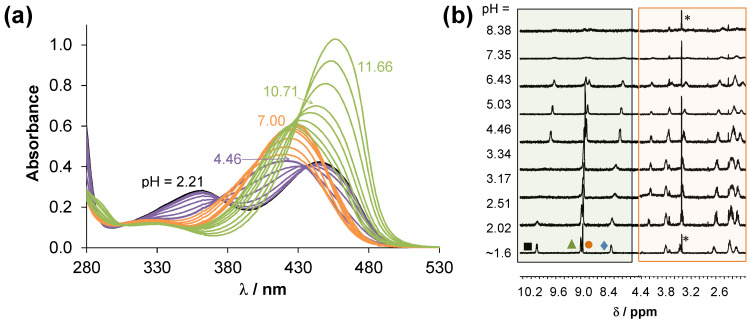
(**a**) UV-vis absorption spectra of the Zn(II)-HQNO_2_-L-Pro (1:2) system at various pH values. {*c*_ligand_ = 40 μM; *c*_Zn(II)_ = 20 μM. (**b**) ^1^H NMR spectra recorded for the same system. Peak assignation is shown in Figure S9 and * denotes the solvent peak. {*c*_ligand_ = 500 μM; *c*_Zn(II)_ = 250 μM; *I* = 0.10 M KCl; *t* = 25.0 °C; 10% (*v*/*v*) D_2_O/H_2_O}.

Complexation of HQNO_2_-L-Pro with Zn(II) was studied by UV-vis and ^1^H NMR spectroscopy (Figure 6). Comparing the UV-vis spectra recorded for the ligand in the presence and in the absence of Zn(II) with increasing pH, complex formation was evident from the UV-vis spectral changes at pH < ~8; however, in the basic pH range the spectra resembled more and more those of the free ligand. Based on the spectra recorded at pH < 8, formation constants for species [Zn(HL)]^+^ and [Zn(HL)_2_] were determined (Table 2). However, these constants suggest that the dissociation of the complexes should not take place in the basic pH range.

^1^H NMR spectra confirm the complex formation in the whole pH range (Figure 6b). Between pH 2 and 4 a strong line broadening is seen, but only in the aromatic region. The mono–ligand complex [Zn(HL)]^+^ and the unbound ligand co-exist in the solution in this pH range, and the ligand-exchange rate seems to be fairly fast on the NMR timescale. Most probably in [Zn(HL)]^+^, the ligand coordinates via the (N,O) donor set of the 8-hydroxyquinoline moiety, and that is why the peaks of the protons of the proline residue are not broadened (only shifted a little). Between 4 and 6.4, sharp resonance signals are seen most likely due to the presence of the sole species [Zn(HL)_2_], although at pH > 7 again broad intensity lines are observed both in the aromatic and aliphatic regions. The signal loss is definitely not due to precipitation. If the complex dissociated completely, the signal of the unbound ligand would appear. Thus, we suggest that there is a structural rearrangement in the complex and the proline moiety coordinates to the Zn(II) in the basic pH. 

**Figure 7:**
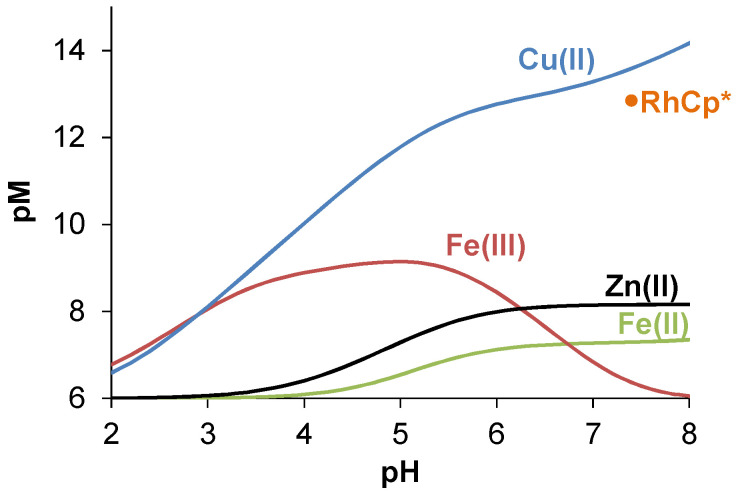
pM values calculated on the basis of the determined stability constants (Table 2) plotted against the pH. pM = −log ([M]+[M_i_(OH)_j_]) (Fe(III): i, j = 1, 1; i, j = 1, 2; i, j = 2, 2; i, j = 3, 4; Fe(II): i, j = 1, 1; i, j = 1, 2; i, j = 1, 3) represents the unbound metal fraction under the given conditions. The value computed for the RhCp* complex is also shown at pH 7.4 (see the conditional stability constant in Section 2.4). {*c*_ligand_ = 10 μM; *c*_M_ = 1 μM; *I* = 0.10 M KCl; *t* = 25.0 °C}.

Comparing the stability constants of the complexes with the studied essential metal ions (Table 2), it can be concluded that, in general, the Cu(II) and Fe(III) complexes possess the highest constants, while Fe(II) and Zn(II) complexes have lower values. On the other hand, different types of complexes are formed, and these metal ions have different affinity towards the hydroxide ions; consequently, based on these constants, we cannot judge the metal ion preferences of HQNO_2_-L-Pro. Therefore, pM values were computed at various pH values for adequate comparison (Figure 7). The higher pM value represents the stronger metal binding ability of the ligand. It clearly shows that at e.g., pH 5, the trend for the metal ion binding is: Cu(II) > Fe(III) > Zn(II) > Fe(II), while at pH 7.4 a different picture is seen: Cu(II) > Zn(II) > Fe(II) > Fe(III), due to the strong tendency of Fe(III) to hydrolyse. 

These data suggest that the title ligand might interact with Cu(II) and Zn(II) ions in the biofluids, although its Fe(III) binding capacity is too weak to remove the metal ion from the binding sites of transferrin in the blood serum, whereas it can act as an efficient Fe(II) chelator in the cytosol (see Figures S16 and S17 in the Supplementary Information for more details). 

### 2.4. Solution Speciation of the RhCp* and RuCym Complexes of HQNO_2_-L-Pro

**Figure 8:**
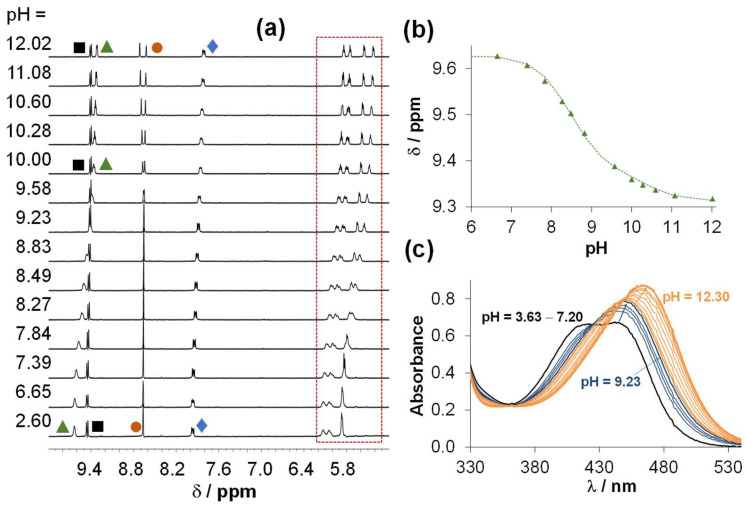
(**a**) Downfield region of the ^1^H NMR spectra recorded for the RuCym complex of HQNO_2_-L-Pro in the pH range 2–12 in 10% (*v*/*v*) D_2_O/H_2_O and (**b**) the chemical shift of CH(2) protons plotted against the pH. Peak assignation is shown in Figure S9 and the framed peaks belong to the *p*-cymene aromatic CH protons. (**c**) UV-vis spectra of the same complex at different pH values. {*c*_complex_ = 500 μM (^1^H NMR) and 50 μM (UV-vis); *I* = 0.20 M KNO_3_; *l* = 1 cm (UV-vis); *t* = 25.0 °C}.

The stability of the isolated RhCp* and RuCym complexes in aqueous solution at pH 7.4 was assayed via recording the UV-vis spectra over 72 h (Figure S18). In the first 24 h, no measurable changes were observed for any of the complexes, only the RhCp* complex showed a minor spectral change after 48 h. Then, the behaviour of the complexes was monitored between pH 2 and 12 by ^1^H NMR spectroscopy (see representative spectra of the RuCym complex in Figure 8) in chloride-free medium, as this halide ion can act as a competitor ligand. There was no indication for the dissociation of the complexes (i.e., liberation of unbound ligand or metal precursor) in the studied pH range or even at pH 1 (Figure S19), showing their fairly high stability in solution. 

The complex formation kinetics for the RhCp* and RuCym complexes was also assayed and compared at pH 1 and 4 (Figure S20). UV-vis spectral changes were followed in time after the triaqua complex [M(arene)(H_2_O)_3_]^2+^ (50 μM) and the ligand (50 μM) were mixed in a tandem cuvette. The equilibrium was reached much faster in the case of the RhCp* complex (~5 min at pH 1, ~1 min at pH 4), as compared to the RuCym complex (~7 h at pH 1, ~45 min at pH 4). These results show that despite the similarly high thermodynamic stability of the two studied complexes, a significant difference is observed in the rate of their complex formation processes.

The prominently high stability of the HQNO_2_-L-Pro complexes, which was also observed for the analogous HQCl-L/D-(h)Pro complexes [12,13], hindered the direct determination of the formation constants. Thus, a ligand displacement study was performed spectrophotometrically using 2,2’-bipyridine as the competitor (Figure S21) to determine the stability constant for the RhCp* complex at pH 7.4. The obtained conditional constant log*K*’ = 11.89 ± 0.02 is lower than those of HQCl-L-Pro (12.98 calculated based on [12]) or 8-hydroxyquinoline (12.76 calculated based on [23]) complexes, suggesting a similar coordination mode but a somewhat lower stability. However, this data still indicates such high stability that <1% of dissociation of the HQNO_2_-L-Pro complex is expected at 1 μM concentration. The affinity of this ligand towards RhCp* is comparable to that towards Cu(II), but is much stronger than towards Fe(II) and Fe(III) (Figure 7). This competition approach could not be used for the RuCym complex due to the release of the *p*-cymene upon the addition of the competitor ligand leading to the slow formation of a mixed-ligand complex (Figure S22), similarly to results obtained with analogous complexes in previous reports [12,13,23].

Indeed, the complexes do not dissociate in a wide pH range; however, spectral changes could be observed by both UV-vis and ^1^H NMR spectroscopy in the basic pH range (see Figure 8 for the RuCym complex). Spectral changes in the UV-vis spectra (Figure 8c) reveal two transformation processes. Namely, the deprotonation of the coordinated aqua ligand and the non-coordinating proline nitrogen takes place at pH > 7.5. Two p*K*_a_ values could be computed for both complexes (Table 3) indicating that these proton dissociation processes, which are partly overlapping, take place at higher pH values in the case of the RhCp* complex, in comparison to the RuCym complex. It should be noted that none of the complexes are found as mixed-hydroxido species in aqueous solution at pH 7.4, which are generally considered as kinetically more inert and biologically less active complexes [32].

Based on the collected speciation data, the complex [M(arene)(HL)(H_2_O)]^+^ is suggested to be the sole species in solution at pH 7.4 in the absence of chloride ions, in which the monoanionic ligand coordinates via the (N,O^−^) donor set, and the Pro moiety is in zwitterionic form (N_Pro_H^+^, COO^−^). As chloride ions can efficiently coordinate to these types of half-sandwich organometallic complexes, the water/chloride co-ligand exchange process was also studied by UV-vis spectrophotometry (at pH 7.4 or 6.0), and log*K*’ (H_2_O/Cl^−^) exchange constants were determined (Table 3) based on the spectral changes upon increasing the chloride concentration in the solution. For the RhCp* complex, a higher constant, namely stronger chloride ion affinity, was obtained. Interestingly, lower log*K*’ (H_2_O/Cl^−^) constants of the corresponding complexes of HQCl-L-Pro and 8-hydroxyquinoline (Table 3) were reported [12,23], suggesting a higher positive charge on the metal centre in the complexes of the nitro derivative.

As a next step, the lipophilicity of the complexes (expressed as distribution coefficient, *D*) was characterized at pH 7.4 at three chloride ion concentrations corresponding to different biofluids (4, 24 and 100 mM for nucleus, cytosol and blood, respectively). Log*D*_7.4_ values are presented in Figure 2 (and in Table S1 together with data of HQCl-L-Pro and its complexes). With decreasing chloride concentration, the complexes become more hydrophilic due to the presence of a less and less neutral [M(arene)(HL)(Cl)] complex. Distribution coefficients for the complexes were also determined at pH 5.5 (Table S1). This pH was chosen as the extracellular pH is often lower in the tumor environment. The complexes display similar log*D* values at the tested pH values, since the composition of the complexes remains the same by decreasing the pH to 5.5. It can be also concluded that the complexes of the nitro derivative are significantly more hydrophilic than the HQCl-L-Pro complexes. 

The stability of the RhCp* and RuCym complexes of HQNO_2_-L-Pro was also monitored in Roswell Park Memorial Institute (RPMI) medium used for the in vitro cell studies (in the case of Colo cells), and in blood serum using UV-vis and ^1^H NMR spectroscopy. The incubation of the complexes in serum resulted in minor changes of the charge-transfer bands in the region 400–500 nm after 24 h (Figure S23). Meanwhile, the changes were more significant in the case of the RPMI medium, indicating the interaction with medium components based on the recorded ^1^H NMR spectra (Figure S24). Despite these changes, the dissociation of the complexes is not likely as the liberation of the unbound ligand was not observed; thus, the complexes most probably form ternary complexes with the components of the serum or the medium. In order to further study the reactivity of these complexes with bioligands, their interaction with human serum albumin (HSA) and selected oligopeptides (also used as binding models of HSA) was assayed. 

### 2.5. Interaction of the RuCym and RhCp* Complexes of HQNO_2_-L-Pro with HSA

Drugs are often bound to plasma proteins and are also subject to displacement from binding sites by other drugs [33]. HSA is the most abundant serum protein and plays an important role in the transport of various drugs. The bound portion often acts as a temporary reservoir from which the compound can be released, and generally the unbound form exhibits the pharmacological effect [33]. At the same time, endogenous albumin can also be harnessed as a drug delivery carrier, since highly stable HSA-drug adducts preferentially accumulate in solid tumors due to the enhanced permeability and retention effect [34]. Therefore, interaction of the title organometallic complexes and the ligand with HSA were investigated with the combined use of CZE, UV-vis, fluorometry, and ^1^H NMR spectroscopic methods.

First, changes in the UV-vis spectra were followed in time in the complex—HSA (1:1 or 2:1) system, and it was concluded that the equilibrium can be reached within ca. 30 min for the RhCp* complex (Figure S25). Meanwhile, only minor changes of the charge transfer bands were detected with the RuCym complex under the applied condition, however, data reflected a significantly slower process. The ^1^H NMR spectra reveal for both complexes that the interaction is not accompanied by the cleavage of the coordination bond between the metal and HQNO_2_-L-Pro, while the peaks of the RhCp* original complexes are shifted, thus, formation of ternary species is suggested (see spectra for the RhCp* complex in Figure S26). The binding of the RuCym complex to HSA without the release of the bidentate ligand was also confirmed by CZE measurement (Figure S27). Most likely a donor atom of the protein binds at the coordination site of the co-ligand.

According to numerous papers on the binding of half-sandwich organometallic complexes to HSA [35,36], surface exposed histidine (His) side chains are the most feasible coordinating moieties. Therefore, interaction of the complexes with C- and N-terminally protected His-containing oligopeptides, namely Ac-Phe-His-Ala-NH_2_ (FHA), Ac-Ala-His-Ala-NH_2_ (AHA), and with the simple monodentate His model, *N*-methylimidazole (mim) was investigated at pH 7.4. The equilibrium could be reached within 3–10 min in the case of the RhCp* complex on the basis of the UV-vis spectral changes (Figure S28), and similar conditional constants (log*K*’) were computed with these model compounds for the formation of mixed-ligand RhCp* complexes; namely log*K*’ 5.00 ± 0.1, 4.4 ± 0.1, 5.1 ± 0.1 were obtained in the case of FHA, AHA, and mim, respectively. The reaction with the RuCym complex was accompanied by minor spectral changes in the UV-vis spectra (similarly as in the case of HSA), which was however significantly slower. Thus, 24 h incubation time was used and ^1^H NMR spectra were recorded which revealed clearly that the RuCym complex is able to react with these model compounds (Figure S29). Most of the peaks in the NMR spectra belonging to the original complex, the binding model, and their mixed-ligand adduct overlapped, which made the determination of the conditional stability constants difficult. In the case of the oligopeptides, formation of isomers was detected which might be explained by the existence of two chiral centers, namely the metal ion and His-CH_α_. The adduct formation of the RuCym complex with mim was quantitative, hindering the determination of the log*K*’ value (meaning log*K*’ > 5.5). Based on the peak integrals (mainly using the signal of the CH(6) proton) log*K*’ 3.9 ± 0.2, and 5.1 ± 0.2 were obtained for the FHA and AHA adducts, respectively.

**Figure 9:**
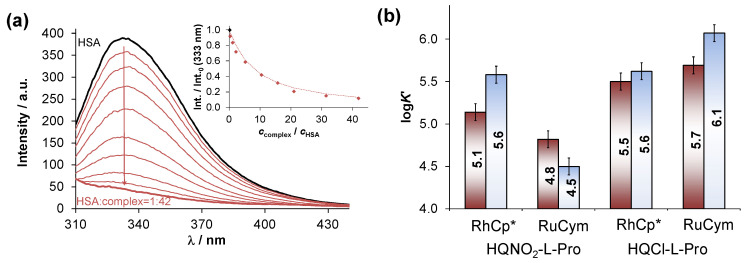
(**a**) Fluorescence emission spectra recorded for HSA upon addition of various amount of RhCp*–HQNO_2_-L-Pro complex using λ_EX_ = 295 nm and 24 h incubation time. Inserted figure shows the intensity (Int.) changes at 333 nm plotted against *c*_complex_/*c*_HSA_ ratio. (**b**) Quenching (log*K*_Q_′, left red bars) and DG marker displacement (log*K*_DG_′, right blue bar) constants of the complexes for their binding to HSA at site I and II, respectively, obtained from spectrofluorometric measurements. Data for HQCl-L-Pro complexes are taken from Ref. [12]. {*c*_HSA_ = 1 µM, *c*_DG_ = 1 µM, *c*_complex_ = 0–42 µM, λ_EX_ = 295 nm (Trp-214) or λ_EX_ = 335 nm (DG); pH = 7.4, (PBS’)}.

All these findings suggest that a surface exposed histidine nitrogen on HSA is also an efficient binder for the studied half-sandwich complexes; however, these model compounds cannot provide reliable insight into the binding interactions taking place in the hydrophobic binding sites of the protein. Therefore, tryptophan (Trp-214) quenching and dansylglycine (DG) site marker displacement experiments were conducted by fluorometry to obtain information about the binding at sites I and II, respectively, using the same approach reported in our former paper [36]. A representative spectral series is shown in Figure 9a for the Trp-214 quenching experiment, upon the addition of the RhCp*–HQNO_2_-L-Pro complex and the computed binding constants (log*K*_Q_’ and log*K*_DG_’) which can be found in Figure 9b, in addition to the corresponding data for the HQCl-L-Pro complexes. It was found that the HQNO_2_-L-Pro complexes can bind to HSA with a lower affinity in comparison to the chloro-analogues, most likely due to their significantly lower distribution coefficients (Table S1). This finding is in line with our suggestion [37] that the albumin binding affinities of RhCp* complexes (including various 8-hydroxyquinoline and 2-picolinate derived complexes as well) correlate with their lipophilicity. 

Additionally, the standalone binding of HQNO_2_-L-Pro was also characterized in the same way, and log*K*_Q_’ = 4.78 ± 0.02 and log*K*_DG_’ = 4.47 ± 0.03 were determined revealing a weaker binding to HSA into both binding pockets, compared to its RhCp* and RuCym complexes. 

### 2.6. Cellular Effects

#### 2.6.1. Inhibitory Effect of HQNO_2_-L-Pro on Organic Anion Transporting Polypeptides

The inhibitory effect of HQNO_2_-L-Pro on the function of organic anion transporting polypeptides (OATPs) was assayed on A431 epidermoid carcinoma cells overexpressing OATPs. OATP1B1 and OATP2B1 are solute carrier-type exchangers [38]. OATP1B1 is specifically expressed in the sinusoidal membrane of hepatocytes [39], while OATP2B1 is ubiquitously expressed, including hepatocytes and enterocytes [40]. Both OATPs are multispecific, i.e., besides endogenous substrate bile acids, bilirubin, and steroid hormones, they also recognize chemically diverse exogenous molecules, including drugs [40,41]. OATP1B1 has a renowned role in the hepatic clearance of statins, which can be affected by drug–drug interactions [41]. Accordingly, the FDA and EMA guidance recommends the investigation of the interaction of a new molecular entity and OATP1B1 [42,43]. OATP2B1 has lower expression levels in hepatocytes compared to OATP1B1; however, it can influence intestinal absorption and blood to brain penetration of drugs [44,45]. OATP1B1 was investigated at physiological pH, while the transport inhibition assay was performed at pH 5.5 in the case of OATP2B1, which is often upregulated in tumors and activated at acidic pH [46].

**Figure 10:**
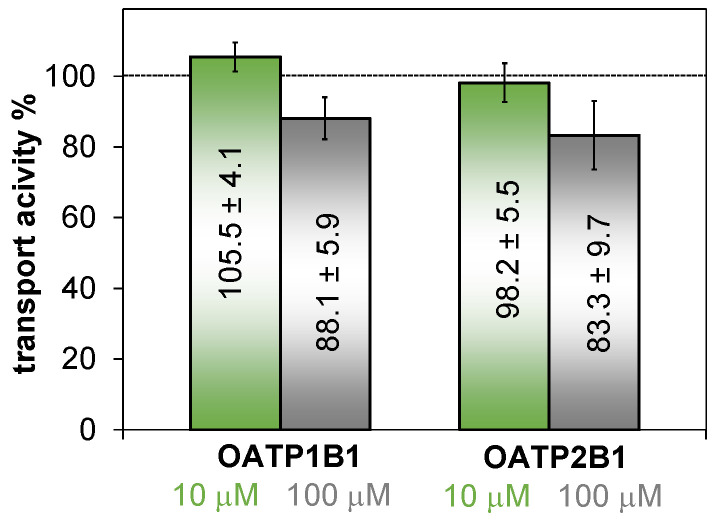
Effect of HQNO_2_-L-Pro on OATP1B1 and OATP2B1 activity. Uptake of disulfopyranine (OATP1B1) and pyranine (OATP2B1) was determined in A431 cells overexpressing these transporters and mock controls, in the absence (100%) or presence of 10 or 100 μM HQNO_2_-L-Pro. OATP-dependent transport was calculated by extracting fluorescence measured in mock transfected cells, and the activity measured in the absence of the tested compound set to 100%. Average (n = 3) ± SD values are shown.

In order to investigate whether HQNO_2_-L-Pro can interact with these drug transporters, we investigated its effect on OATP-mediated uptake of documented fluorescent substrate sulfopyrenes [47]. We found only a minor effect, lower than 20% inhibition of OATP1B1 or OATP2B1 function at 100 μM (Figure 10). In experiments not documented here, the reference inhibitor bromosulfophthalein inhibited the transport of both OATPs. Therefore, we conclude that HQNO_2_-L-Pro will not influence the function of these transporters. Despite the overall negative charge of the molecule at pH 7.4 (or 53% HL^−^ and 47% H_2_L at pH 5.5) the observed effect is weak, most probably the intramolecular hydrogen bonding between the deprotonated hydroxyl group and the protonated proline nitrogen moderates the anionic character of the compound.

#### 2.6.2. Anticancer Activity of the HQNO_2_-L-Pro and Its Half-Sandwich Organometallic Complexes

**Figure 11:**
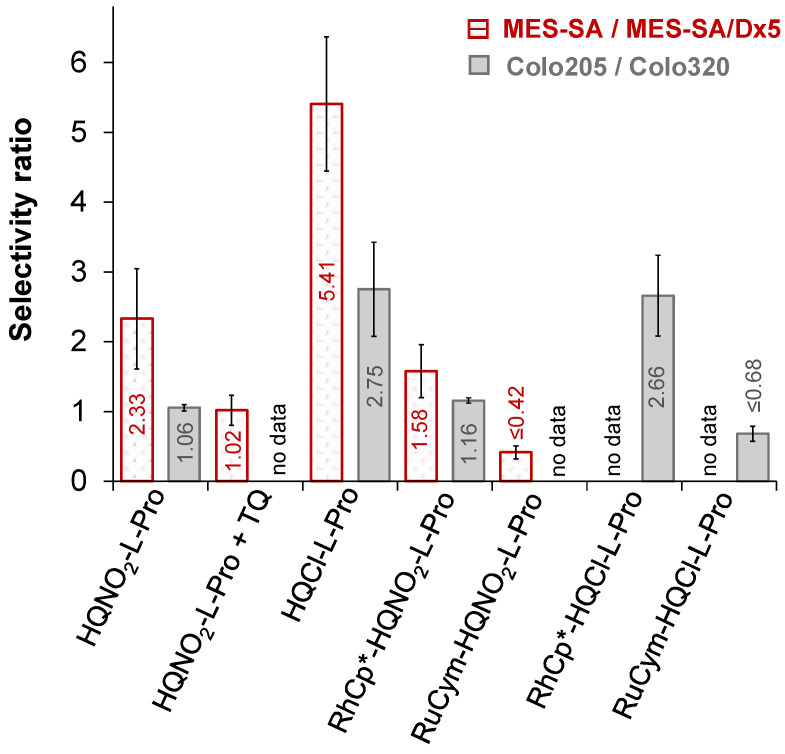
Selectivity ratios calculated on the basis of the IC_50_ values in Table 4: IC_50_ (MES-SA)/IC_50_ (MES-SA/Dx5) (left, dotted columns) and IC_50_ (Colo205)/IC_50_ (Colo320) (right, grey columns) for the ligands HQNO_2_-L-Pro, HQCl-L-Pro and their RhCp* and RuCym complexes. Data for HQCl-L-Pro are taken from Ref. [12].

The in vitro cytotoxicity of HQNO_2_-L-Pro and its RuCym and RhCp* complexes was assayed in two chemosensitive and resistant cell pairs to reveal their potential MDR selective toxicity. On the one hand, the parental human uterine sarcoma MES-SA cell line and its P-gp positive MDR derivative MES-SA/Dx5 cell line were applied; on the other hand, the human colon adenocarcinoma Colo205 cell line and its doxorubicin-resistant counterpart, the Colo320 cell line, were involved. The determined IC_50_ values using 72 h (Colo205 and Colo320) or 144 h incubation time (MES-SA and MES-SA/Dx5) are shown in Table 4. Doxorubicin was used as a control, and IC_50_ values obtained for the analogous HQCl-L-Pro compounds are also shown for comparison. The ligand HQNO_2_-L-Pro showed low-to-moderate standalone cytotoxic activity in the tested cancer cell lines (IC_50_ = 32.4–226 μM) and was significantly less toxic than the chlorine analogue, especially in the MES-SA cells (Table 4). The lower cytotoxicity of HQNO_2_-L-Pro is most likely due to its very strong hydrophilic features. Selectivity ratios were calculated as the quotient of the IC_50_ values obtained in the sensitive and the resistant cells (Figure 11), which reveal selectivity ratios > 1 for both ligands as they exhibited an increased toxicity in the MES-SA/Dx5 and the Colo320 cells in comparison to their chemo-naive counterpart. Therefore, HQNO_2_-L-Pro as well as HQCl-L-Pro are considered to possess a weak MDR selective toxicity. Preferential toxicity of HQNO_2_-L-Pro in MES-SA/Dx5 was abrogated in the presence of the P-gp inhibitor Tariquidar (TQ) (Table 4, Figure 11), revealing the critical contribution of this membrane efflux pump in the MDR selective toxicity. 

The RuCym complex of HQNO_2_-L-Pro exhibited much lower cytotoxicity than the ligand in both adenocarcinoma cell lines and in MES-SA/Dx5 cells, while it was more toxic against the MES-SA cells. However, its selectivity ratios are lower than those of the ligand. The diminished activity was also found for the analogous RuCym complexes of 8-hydroxyquinoline, HQCl-L-Pro, HQCl-D-Pro, and HQCl-D-hPro [12,13,23], most likely due to the arene loss of the complexes in the biofluids upon the interaction with competitor endogenous ligands. Since the release of the *p*-cymene ligand leads to easier oxidation of the metal center, and the forming Ru(III) complex is most probably kinetically more inert, this can hinder the interaction of biological targets. Meanwhile, the RhCp* complex of HQNO_2_-L-Pro is more cytotoxic and possesses higher selectivity ratios than the RuCym complex. It should be noted that its cytotoxicity is comparable to the standalone activity of the ligand.

To reveal the impact of the Fe(III), Cu(II), and Zn(II) ions on the cytotoxicity and MDR selective toxicity of HQNO_2_-L-Pro, it was co-administered with metal salts, and IC_50_ values were also determined in MES-SA and MES-SA/Dx5 cells (Table 4). Complex formation resulted in selective modulation of the toxicity. While the addition of Zn(II) increased the IC_50_ values (except in MES-SA cells), complexation with Cu(II) and Fe(III) decreased them, however the selectivity was also decreased. Based on the determined stability constants, the Cu(II) complexes are present in their bis–ligand forms, while Fe(III) forms tris–ligand complexes in the concentration range of the IC_50_ values. These species are neutral, unlike the negatively charged and zwitterionic ligand, which might be an advantageous property for the uptake process. This effect is less pronounced for Zn(II) as it forms much less stable complexes with HQNO_2_-L-Pro than the other two metal ions.

#### 2.6.3. Antibacterial Activity of the HQNO_2_-L-Pro and Its Half-Sandwich Organometallic Complexes

Half-sandwich cytostatic complexes of RuCym and RhCp* formed with (N,N) donor bearing bidentate monosaccharide ligands were reported to possess antimicrobial activity among Gram-positive bacteria including multiresistant strains [48]. Herein, the antibacterial activity of HQNO_2_-L-Pro and its RuCym and RhCp* complexes was also studied on the Gram-positive *Staphylococcus aureus* and *Enterococcus faecalis* and the Gram-negative *Escherichia coli* and *Klebsiella pneumoniae* strains. Tested compounds had no activity on these bacterial strains (minimum inhibitory concentration (MIC) > 100 μM), except for the RhCp* complex, which displayed a moderate antibacterial effect against the methicillin-resistant *S. aureus* (MRSA) strain (MIC: 50 µM), which is a human pathogen responsible for several difficult-to-treat hospital-acquired infections [49].

## 3. Materials and Methods

### 3.1. Chemicals

All solvents were of analytical grade and used without further purification. [Rh(η^6^-C_5_Me_5_)(μ-Cl)Cl]_2_, [Ru(η^6^-*p*-cymene)(μ-Cl)Cl]_2_, 5-nitro-8-hydroxyquinoline, L-proline, aqueous formaldehyde (30% (*v*/*v*)), bpy, tetrabutylammonium nitrate (TBAN), KH-phthalate, *n*-octanol, D_2_O, doxorubicin, 2-(N-morpholino)ethanesulfonic acid (MES), human serum, Dulbecco’s Modified Eagle Medium (DMEM) and RPMI media, HSA (A8763, essentially globulin free), DG, 1-methylimidazole, 4,4-dimethyl-4-silapentane-1-sulfonic acid (DSS) were Sigma-Aldrich (Darmstadt, Germany) products and were used without further purification. Ac-Ala-His-Ala-NH_2_ (AHA) and Ac-Phe-His-Ala-NH_2_ (FHA) peptides were purchased from GenScript (Piscataway, NJ, USA). HQCl-L-Pro was prepared according to our former report [12]. NaH_2_PO_4_, Na_2_HPO_4_, KH_2_PO_4_, KCl, KNO_3_, NaCl, KOH, HNO_3_, ethanol, ethyl acetate, dichloromethane, *n*-hexane, diethyl-ether, and methanol were purchased from Molar Chemicals (Halásztelek, Hungary) or VWR (Budapest, Hungary).

### 3.2. Stock Solutions and Sample Preparation

Stock and sample solutions were prepared using Milli-Q water. The aqueous stock solutions of [RhCp*(H_2_O)_3_](NO_3_)_2_ and [RuCym(H_2_O)_3_](NO_3_)_2_ were obtained by dissolving an exact amount of the dimeric precursor in water, followed by the addition of equivalent amounts of AgNO_3_, and AgCl precipitate was filtered off. pH-Potentiometric measurements were performed to obtain the exact concentration of the precursor, ligand, and peptide stock solutions. For this purpose, Hyperquad2013 was used [50], as it is reported in our former works [12,13,14]. Stock solutions of RhCp* and RuCym complexes of HQNO_2_-L-Pro were prepared by mixing precursor and ligand stock solutions together in 1:1 ratio, or the direct dissolution of the isolated complexes. In the case of RuCym complexes, slightly acidic conditions were used to avoid the loss of the *p*-cymene. FeCl_3_, ZnCl_2_, and anhydrous CuCl_2_ were dissolved in known amount of HCl and in water, respectively, in order to get the Fe(III), Zn(II) and Cu(II) stock solutions. Their concentrations were determined by complexometry via the EDTA complexes. The Fe(II) stock solution was obtained from fine Fe powder dissolved in a known amount of HCl solution, then filtered, stored and used under anaerobic conditions (O_2_ content ≤ 1 ppm) in a laboratory glove box (GP(Campus), Jacomex). The concentration of the Fe(II) stock solution was determined by permanganometric titrations under acidic conditions. Modified phosphate-buffered saline (PBS’) solution was used to prepare HSA stock solutions. PBS’ contains 12 mM Na_2_HPO_4_, 3 mM KH_2_PO_4_, 1.5 mM KCl, and 100.5 mM NaCl; the concentrations of K^+^, Na^+^, and Cl^−^ ions corresponds to the human blood serum. Residual citrate content of HSA was removed by repeated ultrafiltration of the protein stock solution, and its concentration was calculated from its UV absorption: λ_280nm_ (HSA) = 36,850 M^−1^ cm^−1^ [51]. Samples were prepared in buffer solutions to study the interaction with HSA (PBS’), and were incubated for 24 h at 25 °C. Human serum was diluted with PBS’ buffer four times for stability measurements.

### 3.3. Synthesis and Characterization of Ligands and Their Complexes

#### 3.3.1. Synthesis of HQNO_2_-L-Pro

L-Proline (144 mg, 1.26 mmol), 5-nitro-8-hydroxyquinoline (300 mg, 1.58 mmol), aqueous formaldehyde (30%) (150 mg, 0.15 mmol), and methanol (60 mL) were placed in a 100 mL round bottom flask. The mixture was refluxed at 75 °C for 8 h. The solvent was removed by evaporation. To the residue 50 mL of water was added and the formed crystals were filtered and washed with 10 mL of water. The filtrate was evaporated again and crystallized from a mixture of dichloromethane and methanol (20 mL; 3:1). Yield: 366 mg (73%); melting point: 200–201 °C. Specific rotation at 20 °C: [α]^20^_D_ = +292.3 (*c* = 0.2 g/L, in water). Purity was ≥98% as confirmed by NMR spectroscopy (Figures S1 and S2). ESI-MS (methanol, positive): calc. for [M+H^+^] (C_15_H_16_N_3_O_5_): 318.1090 (*m*/*z*) found: 318.1055 (m/z). ^1^H NMR (D_2_O, δ/ppm): 9.88 (d, J = 8.9 Hz, 1H, H(4)), 8.86 (d, J = 5.1 Hz, 1H, H(2)), 8.80 (s, 1H, H(6)), 8.113 (m, J = 8.9 Hz; 5.2 and 5.2 Hz, 1H, H(3)), 4.49 (m, J = 17.9 Hz; 13.2 and 13.1 Hz, 2H, H(9)), 4.09 (m, J = 9.5 Hz; 5.8 and 5.6 Hz, 1H, H(11)), 3.71 (m, 1H, H(14)), 3.32 (m, 1H, H(14)), 2.51 (m, 1H, H(12)), 2.19 (m, 1H, H(12)), 2.11 (m, 1H, H(13)), 1.98 (m, 1H, H(13)). ^13^C NMR (D_2_O, δ/ppm): 173.8 (C(15)), 168.3 (C(8)), 142.3 (C(2)), 141.2 (C(4)), 135.5 (C(6)), 132.7 (C(8a)), 126.5 (C(5)), 125.9 (C(3)), 125.6 (C(4a)), 115.1 (C(7)), 68.7 (C(11)), 54.8 (C(14)), 54.4 (C(9)), 29.1 (C(12)), 23.4 (C(13)).

#### 3.3.2. Synthesis of [RuCym(HQNO_2_-L-Pro)Cl]Cl

HQNO_2_-L-Pro ligand (2.50 mg, 7.83 µmol) and [Ru(η^6^-*p*-cymene)Cl_2_]_2_ (2.40 mg, 3.92 µmol) were dissolved in diluted nitric acid (1 mL, *c* = 0.01 M, pH_0_ ~2) and stirred at room temperature for 24 h, then the solution was evaporated, followed by a dissolution in dichloromethane, and precipitation was carried out with *n*-hexane. The orange solid was filtered out and dried. Yield: 3.81 mg (78%). NMR spectra are shown in the Supplementary Information (Figures S3 and S4). ESI-MS (methanol, positive): calc. for [RuCym(HQNO_2_-L-Pro)H_−1_]^+^ (C_25_H_28_N_3_O_5_Ru): 552.1073 (*m*/*z*) found: 552.1028 (*m*/*z*); RuCym(HQNO_2_-L-Pro)(Cl)H_−1_ (C_25_H_28_ClN_3_O_5_Ru): 587.0761 (*m*/*z*) found: 587.0784 (*m*/*z*) and [RuCym(HQNO_2_-L-Pro)(Cl)]^+^ (C_25_H_29_ClN_3_O_5_Ru): 588.0839 (*m*/*z*) found: 588.0777 (*m*/*z*). ^1^H NMR (D_2_O, δ/ppm): 9.67 (m, 0.45 H, H_lig_(2)), 9.34 (t, J = 8.0 and 7.5 Hz 0.43 H, H_lig_(2)), 9.24 (d, J = 8.7 Hz, 0.10 H, H_lig_(2)), 9.46 (m, 1H, H_lig_(4)), 8.68 (s, 0.46 H, H_lig_(6)), 8.65 (s, 0.21 H, H_lig_(6)), 8.62 (s, 0.22 H, H_lig_(6)), 8.42 (s, 0.08 H, H_lig_(6)), 7.97 (m, J = 9.0 Hz; 5.0 and 5.0 Hz, 0.47 H, H_lig_(3)), 7.88 (m, 0.45 H, H_lig_(3)), 7.60 (m, J = 8.8 Hz; 5.0 and 4.7 Hz, 0.08 H, H_lig_(3)), δ 6.16–5.56 (6.16 (t, J = 6.2 and 6.6 Hz, 0.46 H), 6.08 (m, 0.51 H), 5.60 (m, 0.92 H), 5.89 (d, 0.98 H), 5.86 (d, J = 6.1 Hz, 0.23 H), 5.78 (t, J = 5.7 and 5.6 Hz, 0.48 H), 5.71 (t, J = 4.7 and 4.8 Hz, 0.45 H), 5.69 (d, J = 5.9 Hz, 0.09 H), 5.56 (d, J = 5.9 Hz, 0.08 H); H_Cym_(C2 & C3 and C5 & C6)), 4.65 (d, J = 13.5 Hz, 0.35 H, H_lig_(9)), 4.57 (m, 0.80 H, H_lig_(9)), 4.49 (d, J = 13.3 Hz, 0.27 H, H_lig_(9)), 4.38 (m, J = 9.4 Hz; 6.0 and 6.2 Hz, 0.24 H, H_lig_(11)), 4.32 (t, J = 6.9 and 8.4 Hz, 0.06 H, H_lig_(11)), 4.22 (m, 0.54 H, H_lig_(11)), 4.14 (m, 0.29 H, H_lig_(11)), 3.92 (m, 0.10 H, H_lig_(14)), 3.75 (m, 0.30 H, H_lig_(14)), 3.64 (m, 0.36 H, H_lig_(14)), 3.56 (m, 0.40 H, H_lig_(14)), 3.45 (m, 0.22 H, H_lig_(14)), 3.31 (m, 0.89 H, H_lig_(14)), 2.76 (m, 1 H, H_Cym_(C7)), 2.54 (m, 1 H, H_lig_(12)), 2.26 (d, J = 4.9 Hz, 2.78 H, H_Cym_(C10)), 2.07 (s, 0.29 H, H_Cym_(C10)), 2.18 (m, 2.08 H, H_lig_(12, 13)), 2.02 (m, 1.08 H, H_lig_(13)), 1.13 (m, 5.51 H, H_Cym_(C8, C9)), 0.89 (dd, ^2^J = 24.6 Hz, ^3^J = 6.9 Hz, 0.51 H, H_Cym_(C8, C9)). ^13^C NMR (D_2_O, δ/ppm): 175.1; 175.0 (C(15)), 172.88 (C(8)), 153.3; 153.2; 152.6; 152.5 (C(2)), δ 142.6; 142.4 (C(8a)) 136.6; 135.5 (C(4)), 133.5; 133.3; 133.0 (C(6)), 129.5; 129.4 (C(5)), 127.3; 127.3; 127.2 (C(3)), 126.1; 126.0; 125.9; 125.9 (C(C4a)), 113.9; 113.8; 113.7 (C(7)), 102.4; 102.2 (C(C4)), 100.2; 100.1; 99.9 (C(C1)), 83.9; 83.5; 83.3; 83.1; 82.9; 82.9; 82.6; 82.3; 82.3; 81.6; 81.5; 80.7; 80.6; 80.3 (C(C2 & C3 and C5 & C6)), 68.3; 68.1; 68.0; 67.8 (C(11)), 54.8; 54.7; 54.2; 54.0; 53.6; 53.0 (C(14 & 9)), 30.7; 30.7; 30.6; 30.4 (C(C7)), 28.9; 28.9; 28.7; 28.5 (C(12)), 23.9; 23.3; 23.1; 22.9 (C(13)), 21.3; 21.2; 21.2; 21.1; 20.9 (C(C8 & C9)), 18.1; 18.0; 17.9; 17.6 (C(C10)).

#### 3.3.3. Synthesis of [RhCp*(HQNO_2_-L-Pro)Cl]Cl

HQNO_2_-L-Pro ligand (2.50 mg, 7.83 µmol) and [Rh(η^5^-C_5_Me_5_)Cl_2_]_2_ (2.42 mg, 3.92 µmol) were dissolved in water (1 mL) and stirred at room temperature for 24 h. Notably, the starting pH was acidic (pH ~3) due to the complexation in which protons are released. Then, the solution was evaporated, followed by dissolution in methanol, and precipitation was carried out with diethyl-ether. The orange solid was filtered out and dried. Yield: 3.44 mg (70%). NMR spectra are shown in the Supplementary Information (Figures S5 and S6). ESI-MS (methanol, positive): calc. for [RhCp*(HQNO_2_-L-Pro)H_−1_]^+^ (C_25_H_29_N_3_O_5_Rh): 554.1163 (*m*/*z*) found: 554.1113 (*m*/*z*) and [RhCp*(HQNO_2_-L-Pro)(Cl)]^+^ (C_25_H_30_ClN_3_O_5_Rh): 590.0929 (*m*/*z*) found 590.0850 (*m*/*z*). ^1^H NMR (DMSO-*d*_6_, δ/ppm): 9.51 (d, J = 8.9 Hz, 1 H, H_lig_(4)), 9.19 (d, J = 4.8 Hz, 1 H, H_lig_(2)), 8.90 (s, 0.60 H, H_lig_(6)), 8.84 (s, 0.40 H, H_lig_(6)), 8.13 (dd, J = 8.9; 4.9 Hz 1 H, H_lig_(3)), 4.76 – 4.52 (3 H, H_lig_(9 & 11)), 3.62 (m, 2 H, H_lig_(14)), 2.56 (m, 1 H, H_lig_(12)), 2.18 (m, 2H, H_lig_(12, 13)), 1.98 (m, 1H, H_lig_(13)), 1.86 (s, 7H, HC_5_Me_5_(CH_3_)), 1.85 (s, 8H, HC_5_Me_5_(CH_3_)). ^13^C NMR (DMSO-*d*_6_, δ/ppm): 174.9 (C(15)), 174.6 (C(15)), 170.94 (C(8)), 170.8 (C(8)), 145.0 (C(2)), 143.7 (C(8a)), 143.4 (C(8a)), 135.1 (C(6)), 134.8 (C(6)), 133.9 (C(4)), 127.9 (C(5)), 127.8 (C(3)), 127.7 (C(3)), 126.1 (C(4a)), 125.9 (C(4a)), 115.2 (C(7)), 94.8 (C(C5)), 94.8 (C(C_5_)), 67.1 (C(11)), 66.6 (C(11)), 53.9 (C(14)), 53.7 (C(14)), 53.0 (C(9)), 51.9 (C(9)), 28.6 (C(12)), 28.5 (C(12)), 23.3 (C(13)), 23.2 (C(13)), 9.0 (C(Me_5_)).

### 3.4. Electrospray Mass Spectrometry

A Waters Q-TOF Premier (Micromass MS Technologies, Manchester, UK) mass spectrometer with an electrospray ion source was used to perform high-resolution (HR) ESI-MS experiments. Samples contained 100 µM compounds (ligand or complex) in methanol (LC-MS grade).

### 3.5. PH-Potentiometric Measurements

The pH-potentiometric measurements were carried out at 25.0 ± 0.1 °C in water in the pH range between 2.0 and 11.5 at a constant ionic strength of 0.2 M KNO_3_. The titrations were performed in a carbonate-free KOH solution (0.20 or 0.10 M). The exact concentrations of HNO_3_, HCl, and KOH solutions were always determined by pH-potentiometric titrations. An Orion 710A pH-meter equipped with a Metrohm combined electrode (type 6.0234.100) and a Metrohm 665 Dosimat burette were used for the measurements. Calibration of the electrode system was carried out by the method suggested by Irving et al. [52]. The average water ionization constant (p*K*_w_) was determined as 13.76 ± 0.01, which is in good agreement with the literature data [53]. In the case of the titrations in 30% (*v*/*v*) DMSO/H_2_O, the calibration of the pH-potentiometric system was conducted as in our former works [54,55]. Samples were degassed by bubbling purified argon through them for about 10 min prior to the measurements, and the inert gas was also passed over the solutions during the titrations. Stoichiometry of the species and their equilibrium constants were obtained similarly to that performed in our previous works [12,13,23], with the use of computer program Hyperquad2013 [50].

### 3.6. UV-Visible Spectrophotometry, Spectrofluorometry, and Circular Dichroism Spectroscopy

An Agilent Cary 8454 diode array spectrophotometer (Santa Clara, CA, USA) was used to obtain UV-vis spectra in the wavelength range 200–1000 nm, while spectra were recorded for the Fe(II)-containing samples using an Avantes (Avantes B.V., Apeldoorn, The Nederlands) AvaSpec-ULS2048CL-EVO spectrometer with an AvaLight-DHc light source and FDP-7UVIR200-2-VAR transmission dip probe. The titrations for the Fe(II)-containing samples were performed in a laboratory glove box (GP(Campus), Jacomex) (O_2_ content ≤ 1 ppm). The path length (ℓ) was 1 cm in most cases (the actual ℓ is always indicated in the legends of the figures). The concentrations of the ligands and complexes were between 40 and 200 µM, and 0.2 M KNO_3_ for the organometallic complexes or 0.1 M KCl for the Fe(II/III), Cu(II), and Zn(II) complexes were used to adjust the constant ionic strength. For HSA containing samples, complex concentrations were 50 µM, and 0.5, and 2 equiv. biomolecules were added for kinetic studies, respectively. For HSA model containing samples, complex concentrations were 50 µM, and the model concentrations were 0–150 µM and 0–500 µM, respectively, and the pH was 7.4 (PBS’). Spectra were always background and baseline corrected. The computer program HypSpec [49] was used to obtain stability constants. Hydrolysis constants of the Fe(III), Fe(II), RuCym, and RhCp* species were included in the speciation models (see Table S4 with the data taken from References [56,57,58,59]). 

CD spectroscopy experiments were performed by a JASCO-J-1500 (ABL&E–JASCO Hungary Ltd., Budapest, Hungary) spectrometer 1 cm optical path. The concentrations of Cu(II) were 500 µM using 1:1 and 1:2 metal-to-ligand ratios in samples prepared in 30% (*v*/*v*) DMSO/H_2_O solvent mixture.

A Fluoromax (Horiba Jobin Yvon, Longjumeau, France) spectrofluorometer was utilized for fluorescence measurements using a 1 cm × 1 cm quartz cuvette. Samples contained 1 µM HSA or DG and 1 µM HSA at pH 7.4 (PBS’), and the complex concentration was varied between 0 and 53 µM. The measurements were carried out on individual samples. Excitation wavelengths were 295 nm for Trp-214 quenching, and 335 nm for the DG displacement studies, respectively. The calculated conditional stability constants for HSA–complex species were obtained using the computer program HypSpec [50]. Calculations always were based on data obtained from at least two independent measurements. Self-absorbance and inner filter effect had to be taken into account [60], and corrections were made as was described in our former works [13,36].

### 3.7. NMR and EPR Spectroscopy

The Bruker Avance III HD Ascend 500 Plus instrument (Billerica, MA, USA) was used for NMR studies. ^1^H NMR spectroscopic measurements were carried out with a WATERGATE water suppression pulse scheme in the presence of 10% (*v*/*v*) D_2_O/H_2_O in most cases. DSS internal standard was added to samples to obtain reference peaks. ^1^H NMR titrations were carried out for the ligand alone, and for the organometallic complexes in the presence of 0.2 M KNO_3_. With the AHA and FHA model peptides, the measurements were performed at pH 7.4 (PBS’) at 0.5 or 1 mM metal complex concentrations, and 1:1, 1:2 or 2:1 peptide-to-metal complex ratios were applied. NMR titrations were also performed for the Zn(II)-ligand systems using 0.1 M KCl ionic strength. The computer program HypSpec [50] was used to obtain equilibrium constants. For ligand and complex characterization, ^13^C NMR spectra were recorded in D_2_O or DMSO-*d*_6_ (10 mM) with the attached proton test (APT) showing CH and CH_3_ as positive peaks, while quaternary C and CH_2_ appear as negative peaks. 

All CW-EPR spectra were recorded at various pH values with a BRUKER EleXsys E500 spectrometer (Billerica, MA, US, microwave frequency 9.45 GHz, microwave power 13 mW, modulation amplitude 5 G, modulation frequency 100 kHz). The solutions for the Cu(II)–HQNO_2_-L-Pro system were prepared in equimolar concentration and at two fold ligand excess in 0.5 mM ligand concentration in 30% (*v*/*v*) DMSO/water. Frozen solution EPR spectra were measured for samples of 0.2 mL in quartz EPR tubes (0.05 mL MeOH was added to each sample to avoid water crystallization upon freezing) and measured in a Dewar containing liquid nitrogen (77 K). EPR spectra were simulated by the EPR program [61]. Rhombic or axial *g*- and *A*-tensors (nuclear spin of Cu = 3/2) were taken into account. Nitrogen splitting was not resolved on the measured spectra. For the description of the linewidth, the orientation dependent α, β and γ parameters were used to set up each component spectra, where α, β and γ defined the linewidths through the equation σ_MI_ = α + β_MI_ + γ_MI_^2^, where M_I_ denotes the magnetic quantum number of the paramagnetic metal ions. 

Dimeric species were also simulated with a module of this EPR software [61]. The principal values and principal orientations of *g*- and *A*-tensors were considered identical, and their relative orientations were characterized by the three Euler angles (α, β and γ). The relative position of the two centers is further described by two polar angles (χ, ψ) which define the position of the connector line between the Cu(II) centers in the frame of *g*_1_. *D* is the dipolar interaction and *J* is the exchange interaction between the two spin centers. For the exchange coupling, an estimation of *J* > 1500 G can be given because under this value a doublet peak originating from this interaction should have been detected under the experimental conditions. The Cu(II)−Cu(II) distances could be calculated from the dipolar coupling by using the point dipole approach. Further details of the simulation process were reported previously [14,62].

Since a natural CuCl_2_ was used for the measurements, both the isotropic and anisotropic spectra were calculated as the sum of the spectra of ^63^Cu and ^65^Cu weighted by their natural abundances. The hyperfine and superhyperfine coupling constants and the relaxation parameters were obtained in field units (Gauss = 10^−4^ T).

### 3.8. Determination of Distribution Coefficients

The traditional shake-flask method was used to obtain distribution coefficients of the ligands as well as of the complexes in *n*-octanol/buffered aqueous solution (20 mM phosphate, pH = 7.40 or 10 mM MES, pH 5.50) at different chloride ion concentrations (4, 24 and 100 mM) using UV-vis spectrophotometry (Agilent Cary 8454 diode array spectrophotometer, Santa Clara, CA, USA) for the analysis. The measurement and data evaluation were performed as described in our former work [12,13].

### 3.9. Cyclic Voltammetry

Cyclic voltammograms were recorded for the Cu(II)–ligand (1:2) and Fe(III)–ligand (1:3) systems at 25.0 ± 0.1 °C at pH 7.4 (10 mM HEPES) using 0.1 M TBAN as the supporting electrolyte. The concentration of the metal ions was 0.5 mM. Measurements were performed on a conventional three-electrode system under argon atmosphere using an Autolab PGSTAT 204 potentiostat/galvanostat monitored with Metrohm’s Nova software (Metrohm Autolab B.V., Utrecht, The Netherlands). Samples were purged for 10 min with argon before recording the cyclic voltammograms. A platinum electrode was used as the working and auxiliary electrode, and Ag/AgCl/3 M KCl as reference electrode. The electrochemical system was calibrated with an aqueous solution of K_3_[Fe(CN)_6_] (*E*_1/2_ = +0.458 V vs. NHE).

### 3.10. Capillary Zone Electrophoresis

An Agilent 7100 capillary electrophoresis system equipped with diode-array UV-vis detector (200–600 nm) was utilized to gain electropherograms. Fused silica capillaries of 48 cm total length with 75 µm inner diameter were used (Agilent Technologies, Santa Clara, CA, USA). The background electrolyte (BGE) was 20 mM phosphate or PBS’ buffer (pH = 7.40), in which the samples were also made. The conditioning process of new capillaries and daily preparation were performed as described formerly [36]. In order to ensure the steady baseline, the capillary was flushed with BGE (2 min) before each run and was rinsed with NaOH (0.1 M; 1.5 min), H_2_O (1.5 min), and then with BGE (2 min) after each run. As post-conditioning, the capillary was also flushed with BGE for 1 min. The sample tray and the capillary cassette were kept at 25 °C. The hydrodynamic injection was used at 50 or 100 mbar for 5 s injection time. For separation, 25 kV voltage was applied to produce a current of ca. 110 μA. The sample run time was set to 6 min. The computer program ChemStation (Agilent) was used to record electropherograms. Electropherograms and spectra of the corresponding peaks were also collected. In order to check the purity of the isolated organometallic complexes, measurements were performed at 0.2 mM compound concentration with the corresponding ligands and precursors as well.

Interaction of the RuCym complex with HSA was studied at constant protein concentration (30 μM) in 20 mM phosphate buffer (pH = 7.40), and the HSA-to-complex ratio varied between 0.04 and 0.83.

### 3.11. OATP Uptake Assay

Cell lines: A431 cells overexpressing OATP1B1 or OATP2B1, and their mock transfected controls were generated and characterized previously [63]. The cell lines were cultured in DMEM (Gibco, Thermofisher Scientific, Waltham, MA, USA), supplemented with 10% fetal bovine serum, 2 mM L-glutamine, 100 μg/mL streptomycin, and 100 units/mL penicillin at 37 °C and 5% CO_2_.

OATP uptake assay: Prior to the experiment, A431 cells (8 × 10^4^/well) were seeded on 96-well plates in 200 μL cell culture medium. After 16–24 h, the cells were washed with 200 μL PBS, three times and pre-incubated with 20–200 μM HQNO_2_-L-Pro, or buffer without HQNO_2_-L-Pro for 5 min at 37 °C in 50 μL HBSS (Hank’s Balanced Salt Solution, pH 7.4; OATP1B1) or uptake buffer (pH 5.5; OATP2B1 [63]). The reaction was started by the addition of disulfopyrene (6,8-dihydroxy-1,3-pyrenedisulfonic acid disodium salt, Sigma, Darmstadt, Germany) at a final concentration of 10 μM (OATP1B1) or pyranine (8-hydroxypyrene-1,3-pyrenetrisulfonic acid trisodium salt, Sigma, Darmstadt, Germany) at a final concentration of 20 μM (OATP2B1), and the cells were further incubated at 37 °C for 10 min (OATP1B1) or 15 min (OATP2B1). The reaction was stopped by removing the supernatant after which the cells were washed three times with ice-cold PBS. Fluorescence (in 200 μL PBS) was determined using an Enspire plate reader (PerkinElmer, Waltham, MA, USA) with excitation/emission wavelengths of 460/510 nm. OATP-dependent transport was calculated by subtracting fluorescence measured in mock transfected cells. Transport activity was calculated based on the fluorescence signal in the absence (100%) of HQNO_2_-L-Pro. Experiments were repeated in at least three biological replicates.

### 3.12. In Vitro Cell Studies: Cell Lines and Culture Conditions and Cytotoxicity Assay

Cell lines: all cell culture reagents were obtained from Sigma-Aldrich; plasticware was purchased from Sarstedt (Nümbrecht, Germany); Human colon Colo205 (chemo-sensitive, ATCC-CCL-222) and Colo320 (Pgp/MDR1-expressing, doxorubicin-resistant, ATCC-CCL-220.1) adenocarcinoma cell lines were purchased from LGC Promochem, Teddington, UK; MES-SA and MES-SA/Dx5 uterine sarcoma cell lines were obtained from ATCC, where they were characterized by DNA fingerprinting. The expression of the Pgp efflux pump of MES-SA/Dx5 was proven by Western blot analysis in our former report [15], and was also reported for the Colo320 cells [64]. The MES-SA and MES-SA/Dx5 cells used in this study were previously engineered to express the fluorescent proteins mCherry and eGFP, respectively, by lentiviral transduction [65].

The colon adenocarcinoma cells were cultured in RPMI 1640 medium supplemented with 10% heat-inactivated fetal bovine serum, 2 mM L-glutamine, 1 mM sodium pyruvate, and 100 mM HEPES. The cells were incubated at 37 °C, in a 5% CO_2_, 95% air atmosphere.

MES-SA cell lines were maintained in DMEM, supplemented with 10% fetal bovine serum, 5 mmol/L glutamine, and 50 units/mL penicillin and streptomycin. Cells were tested and resulted negative for mycoplasma contamination with the MycoAlert mycoplasma detection Kit (Lonza Group, Basel, Switzerland). One week prior to the cytotoxicity assays, Dx5 eGFP cells were selected in 500 nM doxorubicin for one passage to ensure the overexpression of P-glycoprotein.

Cytotoxicity assays: The tested compounds were dissolved in PBS buffer to prepare 5 mM stock solutions, which were diluted in complete culture medium.

In the case of the Colo205 and Colo320 cells, two-fold serial dilutions were prepared in 100 μL medium. The semi-adherent colon adenocarcinoma cells were treated with Trypsin-Versene (EDTA) solution. They were adjusted to a density of 1 × 10^4^ cells in 100 μL of RPMI 1640 medium and were added to each well, with the exception of the medium control wells. The final volume of the wells containing compounds and cells was 200 μL. The plates containing Colo205 and Colo320 cells were incubated at 37 °C for 72 h; at the end of the incubation period, 20 μL of 3-(4,5-dimethyl-2-thiazolyl)-2,5-diphenyl-2H-tetrazolium bromide (MTT) solution (from a stock solution of 5 mg/mL) was added to each well. After incubation at 37 °C for 4 h, 100 μL of sodium dodecyl sulfate (SDS) solution (10% in 0.01 M HCI) was added to each well, and the plates were further incubated at 37 °C overnight. Cell growth was determined by measuring the optical density (OD) at 540/630 nm with a Multiskan EX plate reader (Thermo Labsystems, Cheshire, WA, USA).

In the case of the MES-SA and MES-SA/Dx5 cells, cell suspensions of the fluorescent cell lines were pre-mixed in a 1:1 ratio and seeded in 20 µL medium in a density of 2500 cells/well (1250 cells/well each) in a 384-well plate. After an overnight incubation, a serial dilution of the compounds was added to the plate in a final volume of 60 µL. The P-gp inhibitor tariquidar was added to the cells 15 min prior to the test compounds. After 144 h of incubation, plates were measured for the fluorescent intensity of mCherry (EX/EM: 585 nm/610 nm) and eGFP (EX/EM: 485 nm/510 nm). Cell seeding, aspiration of medium, and addition of drugs were performed by a Hamilton StarLet automated liquid handling robot.

Inhibition of the cell growth (expressed as IC_50_: inhibitory concentration that reduces by 50% the growth of the cells exposed to the tested compounds) was determined from the sigmoid curve where 100 − ((OD_sample_ − OD_medium control_)/(OD_cell control_ − OD_medium control_)) × 100 values were plotted against the logarithm of compound concentrations. Curves for the data obtained on Colo205 and Colo320 cells were fitted by GraphPad Prism software (2021, Graphpad Software, version 7.00, San Diego, CA, USA) using the sigmoidal dose–response model (comparing variable and fixed slopes). From the MES-SA cell data, the IC_50_ values were calculated by our custom program, written by J. Sessler in C#. The p values were calculated by Student’s t-test. The IC_50_ values were always obtained from at least three independent experiments.

### 3.13. Antibacterial Effect: Bacterial Culture and Determination of MIC Values

*Escherichia coli* (ATCC 25922), *Klebsiella pneumoniae* (ATCC 49618) were used as Gram-negative strains; and *Staphylococcus aureus* (ATCC 25923, methicillin-resistant (MRSA)), and *Enterococcus faecalis* (ATCC 29212) strains were applied as Gram-positive bacterial cultures in the experiments. MIC values of the complexes were determined in 96-well plates based on the Clinical and Laboratory Standard Institute guidelines (CLSI guidelines) [66]. The stock solutions of the compounds (dissolved in PBS buffer using 5 mM concentration) were diluted in 100 μL of Mueller Hinton Broth. Then, 10^−4^ dilution of an overnight bacterial culture in 100 μL of medium was added to each well, with the exception of the medium control wells. The plates were further incubated at 37 °C for 18 h; at the end of the incubation period, the MIC values of tested compounds were determined by visual inspection.

## 4. Conclusions

Chemotherapy applied as standard anticancer treatment is often accompanied by serious side effects; moreover, multidrug resistance (MDR) is a growing concern in medical oncology. In order to circumvent MDR, various 8-hydroxyquinolines were developed with increased, rather than decreased, cytotoxicity against Pgp-expressing MDR cells [5,6,7]. This type of compound has rather low solubility in water, and to optimize the drug-likeness properties, well-balanced hydrophilic–lipophilic characteristics are needed. The incorporation of the zwitterionic proline and homoproline in the scaffold of 5-chloro-8-hydroxyquinoline resulted in increased hydrophilicity [12,13], and the exchange of the chloro to nitro substituent in the herein developed HQNO_2_-L-Pro has resulted in even better solubility.

HQNO_2_-L-Pro displayed relatively weak cytotoxicity against the tested cell lines. Still, despite lower toxicity, HQNO_2_-L-Pro proved to be more active against MDR derivatives, indicating that the chemical modifications resulting in improved solubility did not eliminate MDR-selective toxicity. Since complexation with iron (and copper) was shown to be linked to the toxicity and MDR-selective activity of the compounds [5,15], we studied the interaction of this novel compound with Fe(III) and Fe(II) ions by UV-vis spectrophotometric titrations. We found that the resulting complexes have moderate stability and a higher redox potential, as compared to the iron complexes of the non-selective 8-hydroxyquinoline; this likely explains the MDR-selective toxicity of HQNO_2_-L-Pro. HQNO_2_-L-Pro had only a minor impact on the function of OATP1B1 and OATP2B1 organic anionic transporter polypeptides. The compound has significant affinity to Cu(II) and somewhat lower binding ability towards Zn(II) ions, according to the UV-vis, EPR, CD, and ^1^H NMR spectroscopic measurements. The proline residue did not merely impact aqueous solubility, but also resulted in different coordination modes in the case of Cu(II) and Zn(II) complexes, as compared to the typical binding mode via the (O,N) donor set in 8-hydroxyquinolines. Additionally, Rh(η^5^-C_5_Me_5_) and Ru(η^6^-*p*-cymene) complexes of HQNO_2_-L-Pro were prepared and tested for their anticancer activity. Complexation changed the overall charge, size, and albumin binding properties, resulting in improved pharmaceutical properties. Complex formation with Ru(η^6^-*p*-cymene) inactivated the ligand, whereas the Rh(η^5^-C_5_Me_5_) complex exhibited pronounced MDR-selective activity. In summary, both the HQNO_2_-L-Pro ligand and the Rh-complex show good aqueous solubility, and therefore can be considered as good candidates for further biological studies.

## Figures and Tables

**Table 1:** p*K*_a_ values of HQNO_2_-L-Pro determined by various methods. {*t* = 25.0 °C}.

Method	Ionic Strength (*I*)	p*K*_a_ (OH)	p*K*_a_ (N_Pro_H^+^)	*c*_ligand_/μM
pH-potentiometry	0.20 M KNO_3_	5.57 ± 0.09	10.68 ± 0.07	1700
^1^H NMR	0.20 M KNO_3_	5.56 ± 0.03	>10.5	1000
UV-vis	0.20 M KNO_3_	5.50 ± 0.03	10.78 ± 0.03	40
UV-vis	0.10 M KCl	5.43 ± 0.03	10.75 ± 0.03	40

**Table 2:** Formation constants (log*β*) of the Fe(III), Fe(II), Cu(II) and Zn(II) complexes with HQNO_2_-L-Pro determined by UV-vis spectrophotometric titrations. (H_2_L is the neutral form of the ligand, charges of the complexes are omitted for clarity.) {*I* = 0.1 M (KCl); *t* = 25.0 °C}.

log*β*	Fe(III)	Fe(II)	Cu(II)	Zn(II)
[M(HL)]	19.75 ± 0.03	16.52 ± 0.03	19.40 ± 0.03	17.36 ± 0.03
[M(L)]	−	8.39 ± 0.03	14.45 ± 0.03	n.d. ^a^
[M(HL)_2_]	38.76 ± 0.03	32.74 ± 0.03	38.60 ± 0.03	33.71 ± 0.06
[M(HL)(L)]	−	−	31.75 ± 0.03	n.d. ^a^
[M(L)_2_]	−	−	22.99 ± 0.03	n.d. ^a^
[M(HL)_3_]	55.66 ± 0.03	47.60 ± 0.06	−	−

^a^ Data were evaluated at pH < 8.

**Table 3:** p*K*_a_ values of the RhCp* and RuCym complexes of HQNO_2_-L-Pro and the H_2_O/Cl^−^ exchange constants determined (at pH 7.4 for RhCp*, pH 6.0 for RuCym) by UV-vis spectrophotometric titrations. {*I* = 0.2 M (KNO_3_); *t* = 25.0 °C}.

	RhCp* Complex	RuCym Complex
p*K*_a_ (1)	9.33 ± 0.03	8.55 ± 0.03
p*K*_a_ (2)	10.34 ± 0.03	10.21 ± 0.03
log*K*’ (H_2_O/Cl^−^) ^a^	2.07 ± 0.03	1.47 ± 0.01

^a^ log*K*’ (H_2_O/Cl^−^) for RhCp*-HQCl-L-Pro: 1.57 [12], RhCp*-HQ: 1.81 [23], RuCym-HQCl-L-Pro: 1.21 [12], RuCym-HQ: 0.89 [23].

**Table 4:** In vitro cytotoxicity (IC_50_ values in μM) of HQNO_2_-L-Pro and its RuCym and RhCp* complexes in Colo205, Colo320 {72 h exposure}, MES-SA and MES-SA/Dx5 {144 h exposure} cell lines. IC_50_ values obtained for the analogous HQCl-L-Pro compounds are also shown for comparison. IC_50_ values obtained for HQNO_2_-L-Pro in the presence of various equiv. CuCl_2_, ZnCl_2_ and FeCl_3_. Doxorubicin was used as a control.

	IC_50_ (μM)			
	MES-SA	MES-SA/Dx5	Colo205	Colo320
**ligand alone**				
HQNO_2_-L-Pro	226 ± 17	97 ± 29	34 ± 1	32.4 ± 0.4
HQNO_2_-L-Pro + TQ	223 ± 6	219 ± 46	-	-
HQCl-L-Pro	23 ± 4	4.3 ± 0.1	23 ± 3 ^a^	9 ± 2 ^a^
**complexes**				
RuCym-HQNO_2_-L-Pro	166 ± 37	>400	>100	>100
RhCp*-HQNO_2_-L-Pro	167 ± 38	106 ± 8	35 ± 1	29.8 ± 0.2
RuCym-HQCl-L-Pro	-	-	68 ± 11 ^a^	>100 ^a^
RhCp*-HQCl-L-Pro	-	-	26 ± 5 ^a^	10 ± 1 ^a^
**incubation of HQNO_2_-L-Pro with metal salts**				
+1 equiv. Cu(II)	40 ± 7	25 ± 6	-	-
+0.5 equiv. Cu(II)	49 ± 15	35 ± 11	-	-
+1 equiv. Zn(II)	164 ± 24	>400	-	-
+0.5 equiv. Zn(II)	>400	>400	-	-
+1 equiv. Fe(III)	89 ± 9	47 ± 9	-	-
+0.33 equiv. Fe(III)	121 ± 27	53 ± 9	-	-
doxorubicin	0.03 ± 0.01	4 ± 1	0.71 ± 0.02	5.4 ± 0.9

^a^ Data are taken from Ref. [12].

## Data Availability

Authors can confirm that all relevant data are included in the article.

## Supplementary Materials

The supporting information can be downloaded at: https://www.mdpi.com/article/10.3390/ijms24010593/s1. References [12,56,57,58,59,67,68,69,70,71,72,73,74] are cited in the supplementary materials.
