# Supplementary material for: Metal Complexes of a 5-Nitro-8-Hydroxyquinoline-Proline Hybrid with Enhanced Water Solubility Targeting Multidrug Resistant Cancer Cells
Source: Int J Mol Sci. 2022 Dec 29;24(1):593. doi: 10.3390/ijms24010593 (PMC9820345; doi:10.3390/ijms24010593)
Supplement: Supplementary file 1 [file ijms-24-00593-s001.zip › ijms-2097939-supplementary.pdf]

Metal complexes of a 5-nitro-8-hydroxyquinoline-proline hybrid with enhanced water solubility targeting multidrug resistant cancer cells

Tamás Pivarcsik, Vivien Pósa, Hilda Kovács, Nóra V. May, Gabriella Spengler, Szonja P. Pósa, Szilárd Tóth, Zeinab Nezafat Yazdi, Csilla Özvegy-Laczka, Imre Ugrai, István Szatmári, Gergely Szakács and Éva A. Enyedy*

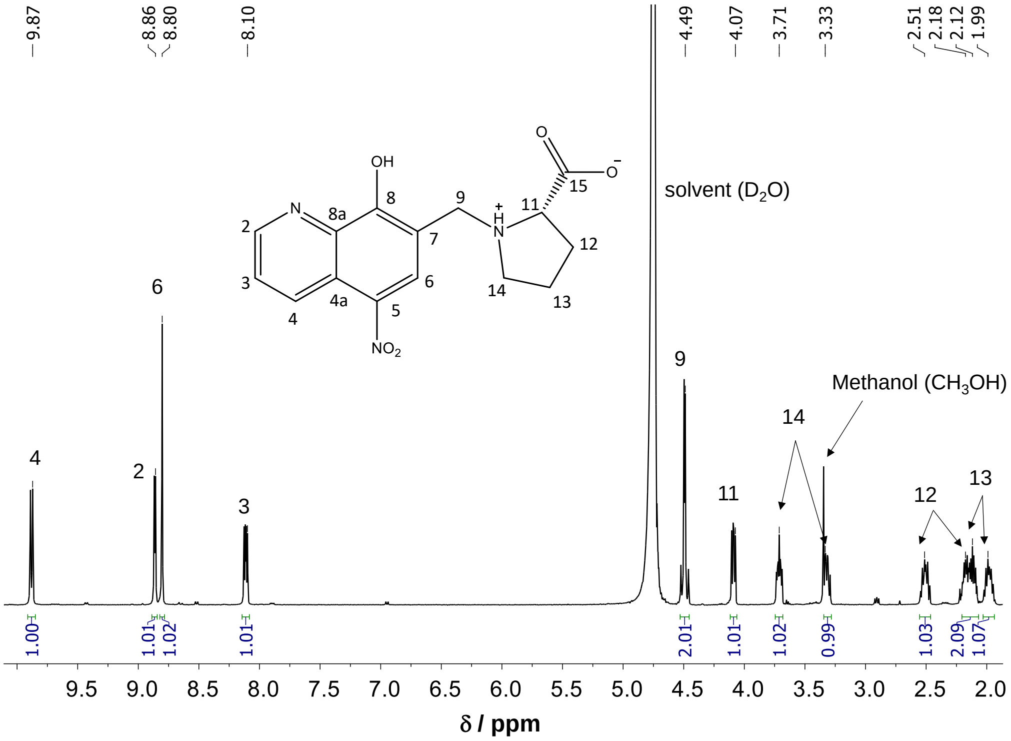

Figure S1. ^{1}H NMR spectrum of HQNO2-L-Pro in D_2O. Inserted structure shows numbering of peaks. {$c_{HQNO2\text{-}L\text{-}Pro}$ = 10 mM; t = 25.0 °C}

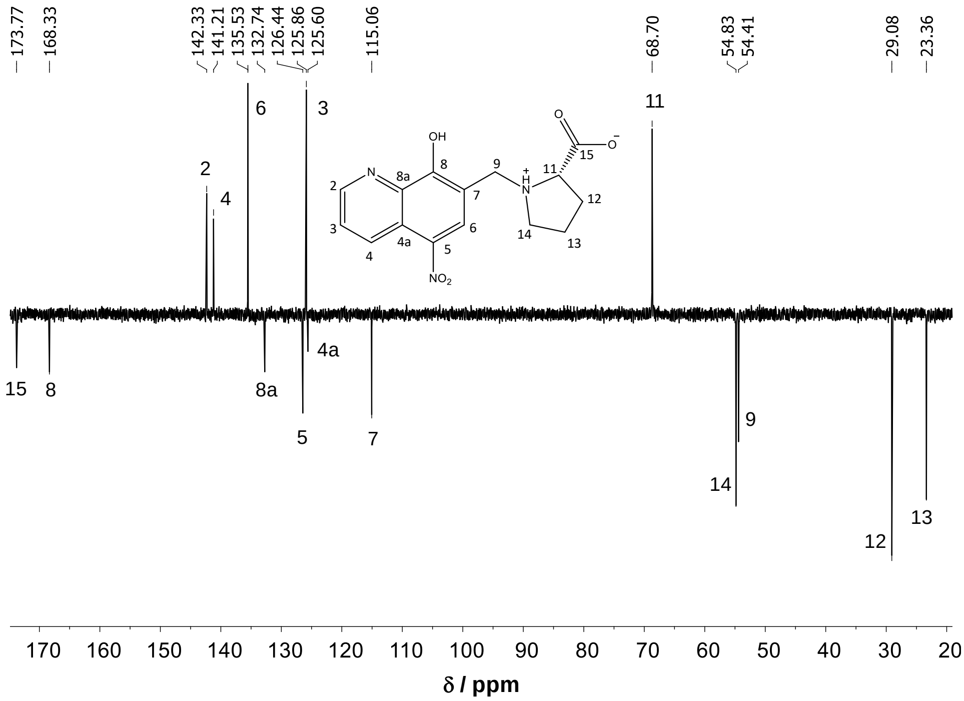

Figure S2. ^{13}C APT NMR spectrum of HQNO$_2$-L-Pro in D_2O. Attached proton test method: CH and CH_3 peaks are positive, C and CH_2 peaks are negative. Inserted structure shows numbering of peaks. {$c_{HQNO2\text{-}L\text{-}Pro}$ = 10 mM; t = 25.0 °C}

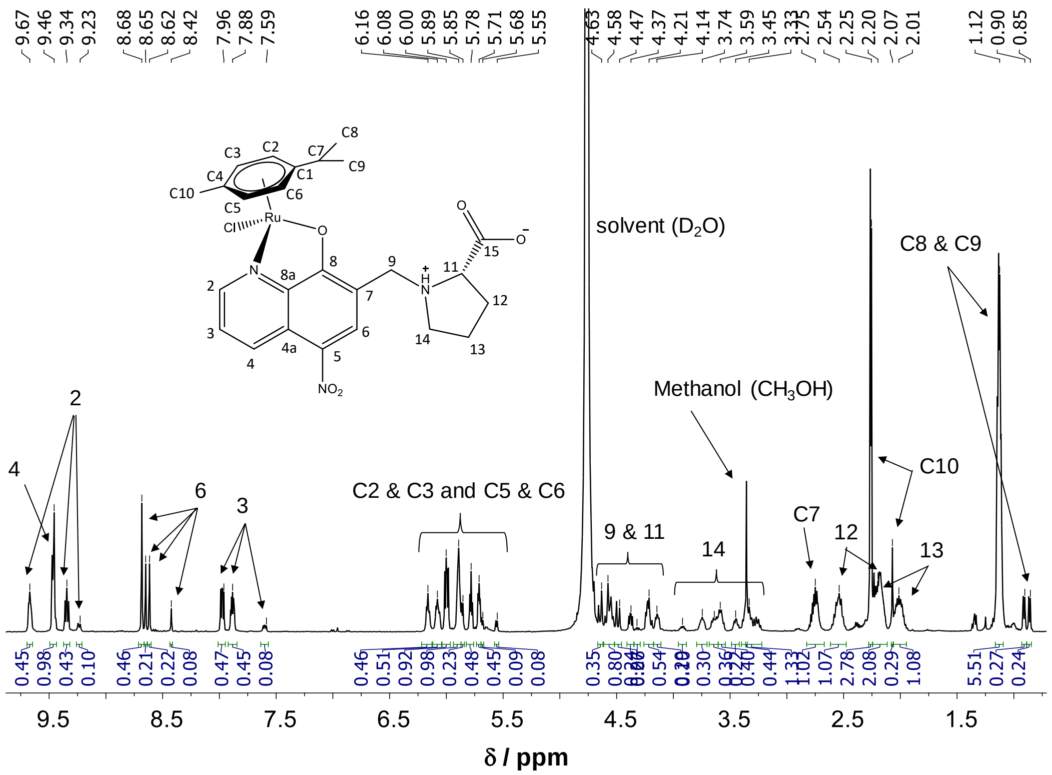

Figure S3. ^{1}H NMR spectrum of [Ru(η^6-*p*-cymene)(HQNO$_2$-L-Pro)Cl]Cl in D_2O. Inserted structure shows numbering of peaks. {$c_{complex}$ = 10 mM; *t* = 25.0 °C}

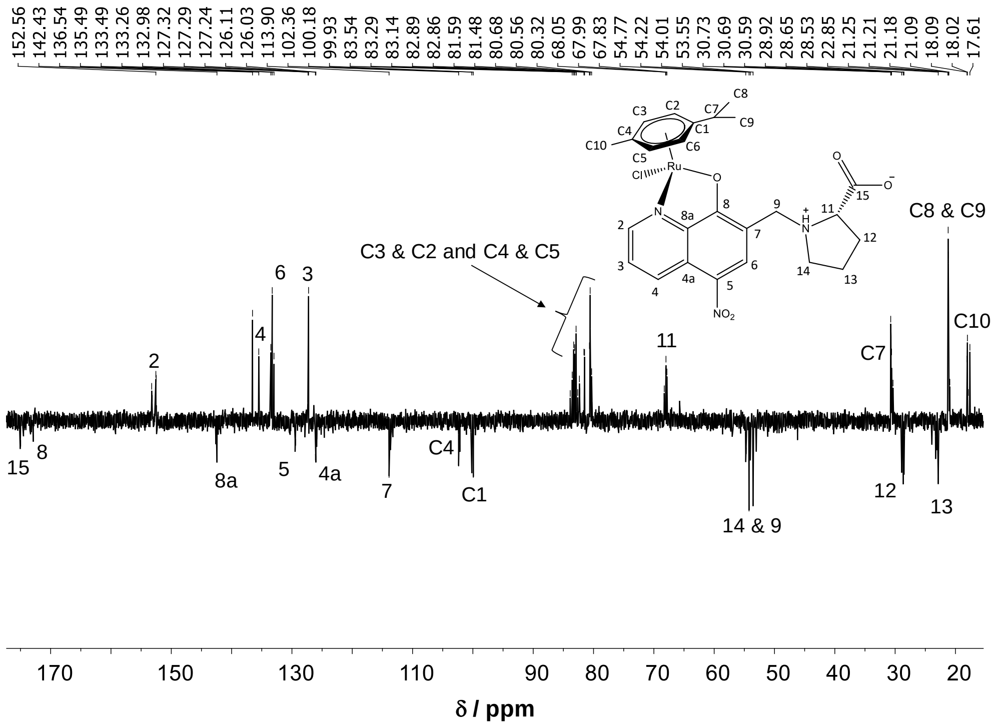

Figure S4. ^{13}C APT NMR spectrum of the [Ru(η^6-*p*-cymene)(HQNO$_2$-L-Pro)Cl]Cl in D_2O. Attached proton test method: CH and CH_3 peaks are positive, C and CH_2 peaks are negative. Inserted structure shows numbering of peaks. {$c_{complex}$ = 10 mM; t = 25.0 °C}

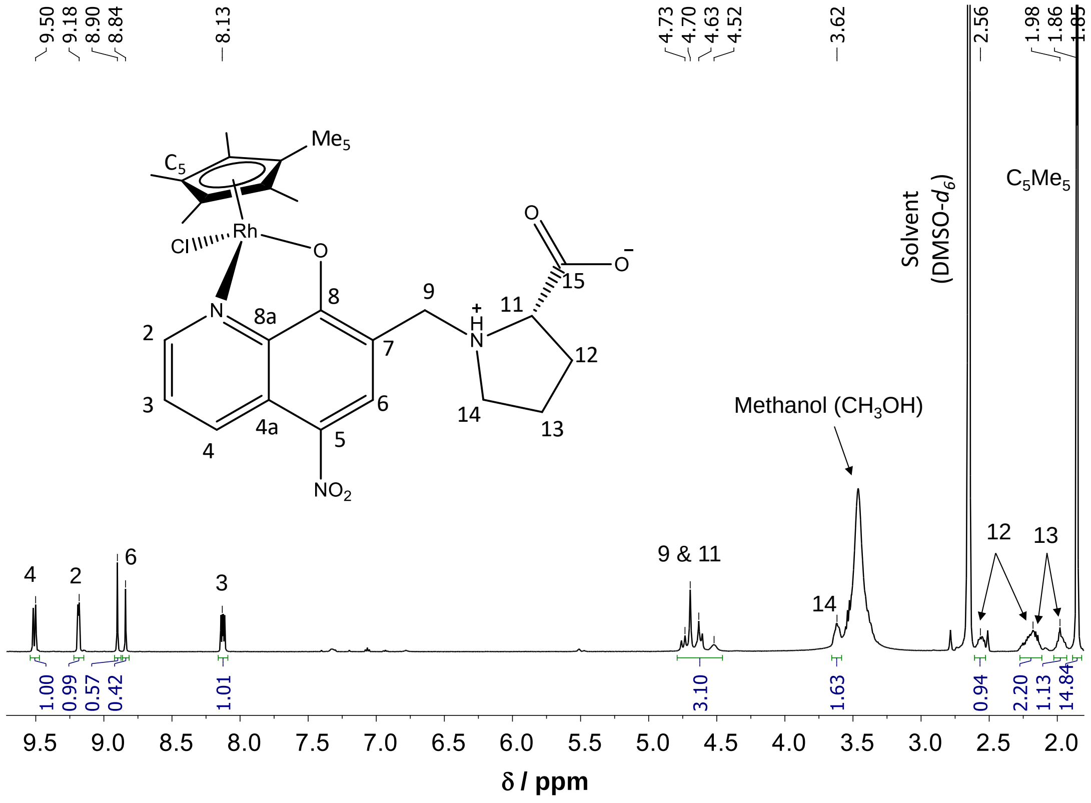

Figure S5. ^1H NMR spectrum of [Rh(η^5-C_5Me_5)(HQNO$_2$-L-Pro)Cl]Cl in DMSO-*d*$_6$. Inserted structure shows numbering of peaks. {$c_{complex}$ = 10 mM; t = 25.0 °C}

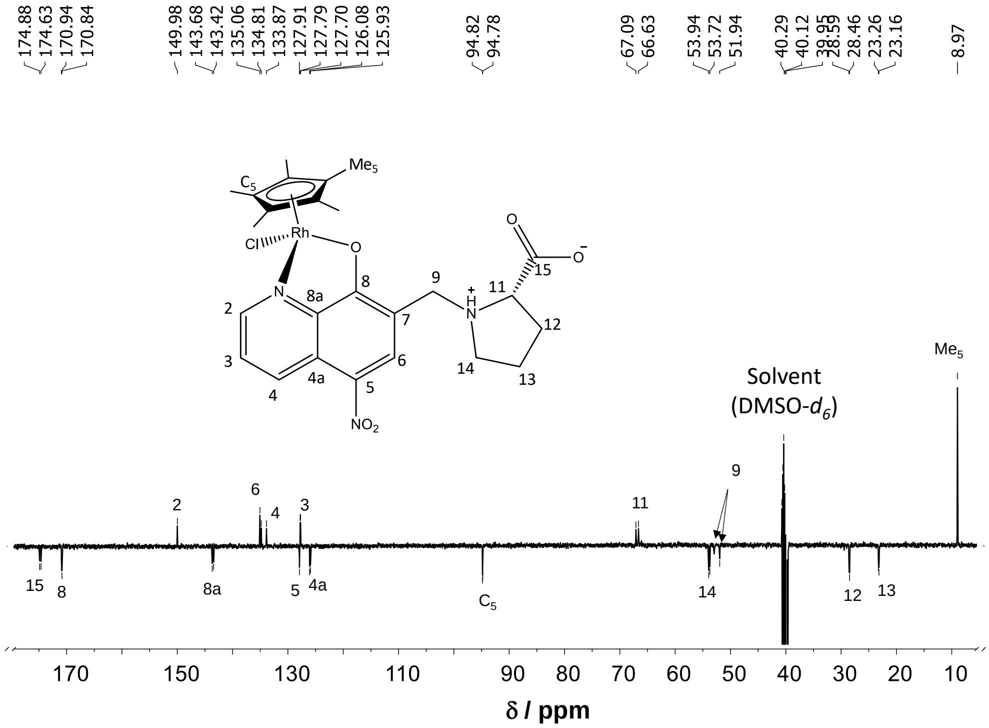

Figure S6. ^{13}C APT NMR spectrum of [Rh(η^5-C_5Me_5)(HQNO$_2$-L-Pro)Cl]Cl in DMSO-d_6. Attached proton test method: CH and CH_3 peaks are positive, C and CH_2 peaks are negative. Inserted structure shows numbering of peaks. (Signal of C7 could not be observed well.) {$c_{complex}$ = 10 mM; t = 25.0 °C}

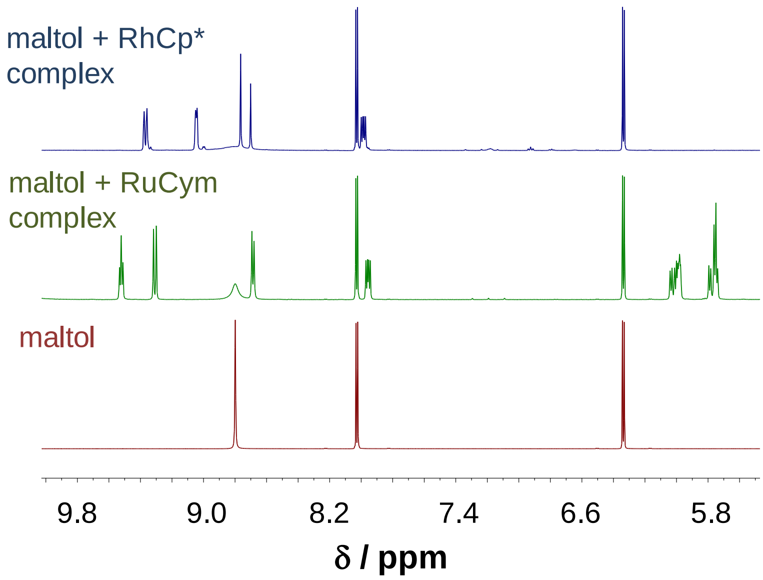

Figure S7. ^{1}H NMR spectrum of maltol, the [Rh(η^5-C_5Me_5)(HQNO$_2$-L-Pro)Cl]Cl and [Ru(η^6-*p*-cymene)(HQNO$_2$-L-Pro)Cl]Cl complexes together with maltol in DMSO-d_6. Based on the integrals the RhCp* complex is found with 2 chloride ions and 4 water molecules, and the RuCym complex with 2 chloride ions and 2 water molecules.

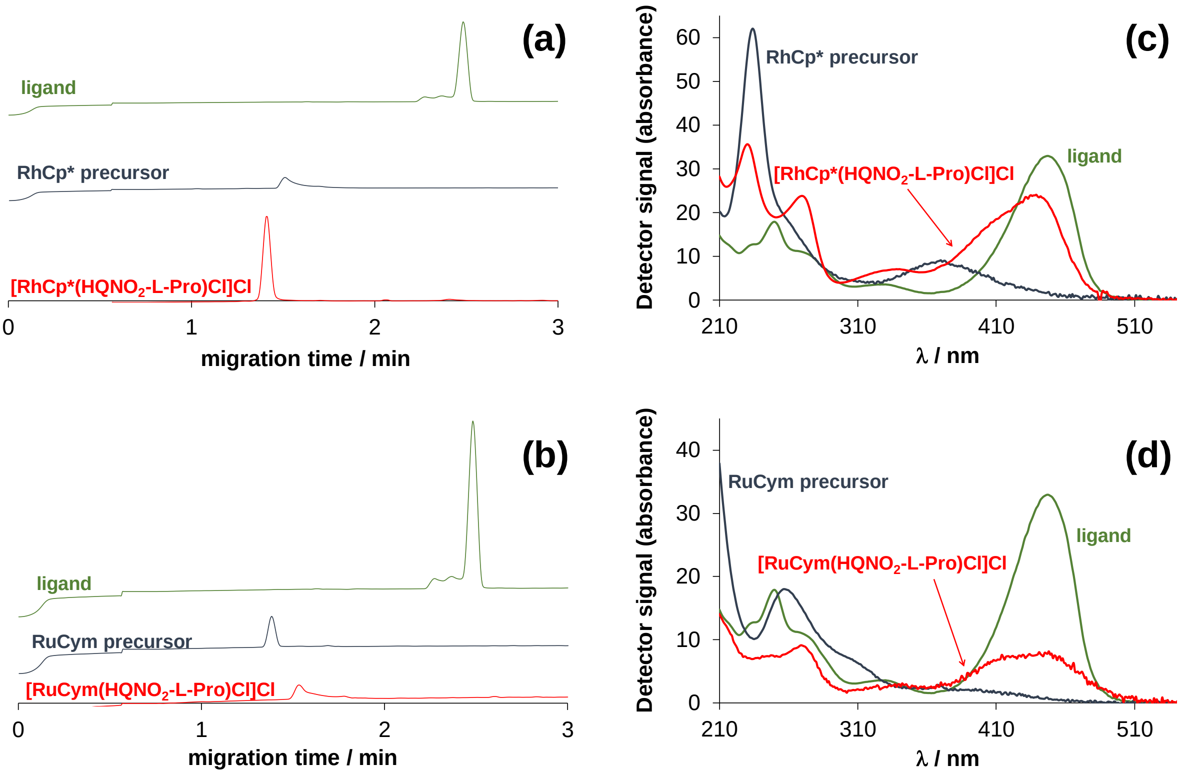

Figure S8. Electropherograms of the ligand alone, the metal precursor and the complex in the case of (a) RhCp* and (b) RuCym. The UV-vis spectrum measured at the peak maximum for the (c) RhCp* and (d) RuCym complex and the spectra of the ligand and the corresponding precursors are also shown. {$c_{complex}$ = 50 – 100 μM; $c_{\text{precursor}}$ = 100 μM; c_{ligand} = 50 μM; pH = 7.40 PBS′ buffer}

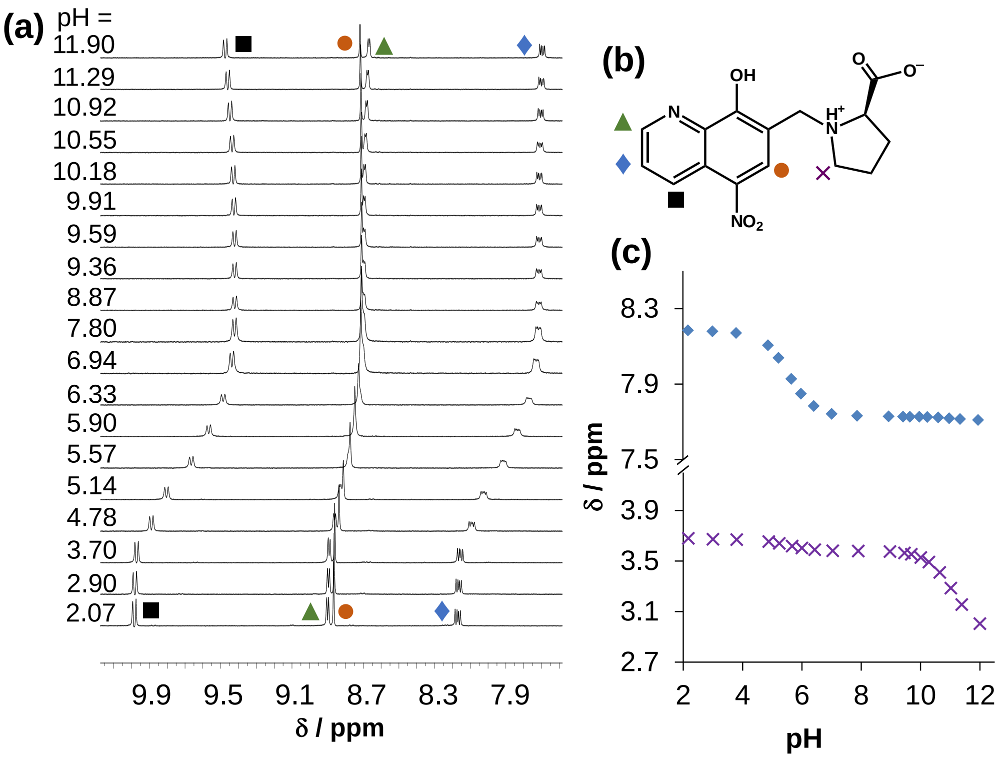

Figure S9. (a) ^{1}H NMR spectra of HQNO$_2$-L-Pro recorded at various pH values in the low-field region. (b) Peak assignation shown with symbols. (c) Chemical shifts of two selected protons (CH(3), CH(14)) plotted against the pH. {c_{ligand} = 1 mM; 10% (v/v) D_2O/H_2O; I = 0.2 M KNO_3; t = 25.0 °C}

Table S1. *n*-Octanol/water distribution coefficients for the ligand HQNO$_2$-L-Pro and HQCl-L-Pro and their RhCp* and RuCym complexes at different chloride ion concentrations: 4 mM, 24 mM and 100 mM at pH = 7.4 (log $D_{7.4}$) and at pH 5.5 (log $D_{5.5}$). {pH = 7.40 (20 mM phosphate buffer); pH = 5.5 (10 mM MES); t = 25.0 °C}

	log $D_{7.4}$			log $D_{5.5}$
	**4 mM**	**24 mM**	**100 mM**	**100 mM**
HQNO$_2$-L-Pro	–1.05 ± 0.14	–0.93 ± 0.18	–0.94 ± 0.10	–0.91 ± 0.01
HQCl-L-Pro [a]	+0.13 ± 0.09	–0.01 ± 0.01	–0.03 ± 0.01	–
RhCp* complex of HQNO$_2$-L-Pro	–0.74 ± 0.13	–0.61 ± 0.01	–0.59 ± 0.01	–0.68 ± 0.01
RuCym complex of HQNO$_2$-L-Pro	–1.28 ± 0.12	–0.29 ± 0.02	+0.06 ± 0.01	–0.01 ± 0.01
RhCp* complex of HQCl-L-Pro [a]	–0.53 ± 0.03	–0.12 ± 0.01	+0.10 ± 0.01	–
RuCym complex of HQCl-L-Pro [a]	–0.36 ± 0.03	+0.09 ± 0.03	+0.46 ± 0.03	–

[a] Data are taken from Ref. [12].

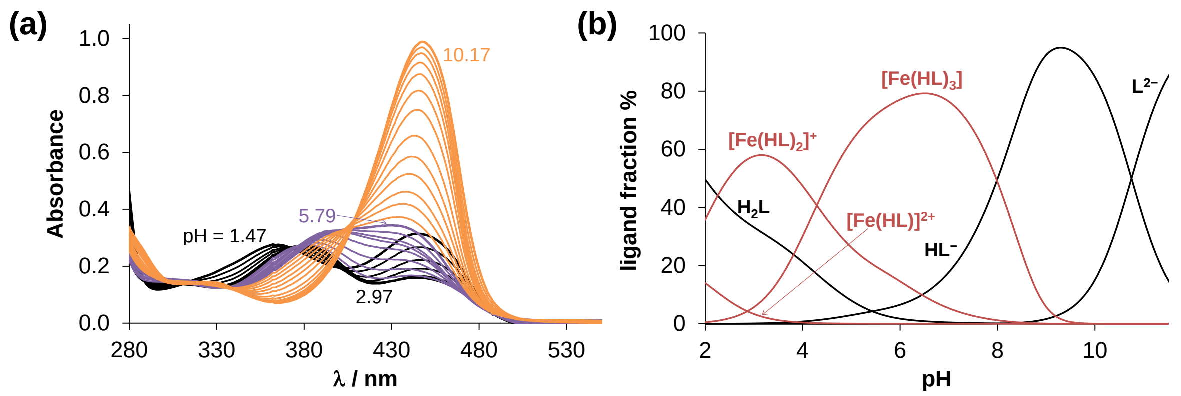

Figure S10. (a) UV-vis spectra recorded for the Fe(III) – $HQNO_2$-L-Pro system in the pH range 1.5 – 10.2 at 1:3 Fe(III):ligand ratio. (b) Concentration distribution curves for the same system calculated on the basis of the stability constants determined. {c_{ligand} = 38 μM; $c_{Fe(III)}$ = 13 μM; I = 0.10 M KCl; l = 1 cm; t = 25.0 °C}

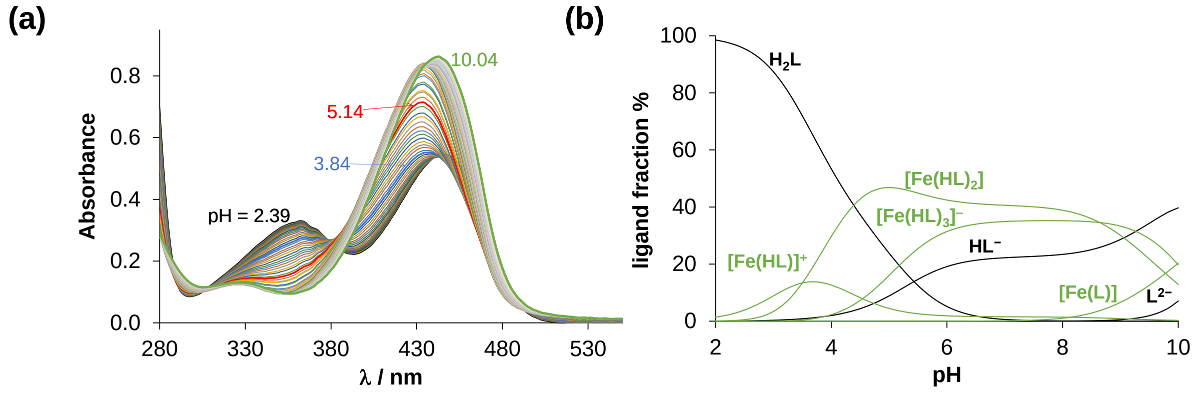

Figure S11. (a) UV-vis spectra recorded for the Fe(II) – $HQNO_2$-L-Pro system in the pH range 2.4 – 10.0 at 1:3 Fe(II):ligand ratio. (b) Concentration distribution curves for the same system calculated on the basis of the stability constants determined. {c_{ligand} = 200 μM; $c_{Fe(III)}$ = 67.4 μM; I = 0.10 M KCl; l = 0.226 cm; t = 25.0 °C}

Table S2. Electrochemical data collected for the iron(III/II) – $HQNO_2$-L-Pro (1:3) system by the cyclic voltammetric measurements. {c_{ligand} =1.5 mM, $c_{Fe(III)}$ = 0.5 mM; pH = 7.40 (10 mM HEPES); t = 25 °C; I = 0.1 M (TBAN)}

scan rate (V/s)	E_c (V)	E_a (V)	ΔE (V)	$E_{1/2}$ (V)	$E_{1/2}$ *vs.* NHE (V)	$\|i_c/i_a\|$
**0.005**	0.039	-0.045	0.084	-0.003	+0.207	0.76
**0.010**	0.041	-0.029	0.070	0.006	+0.216	0.86
**0.015**	0.043	-0.026	0.069	0.008	+0.218	0.88
**0.020**	0.046	-0.033	0.079	0.007	+0.217	0.91
**0.025**	0.046	-0.038	0.085	0.004	+0.214	0.96

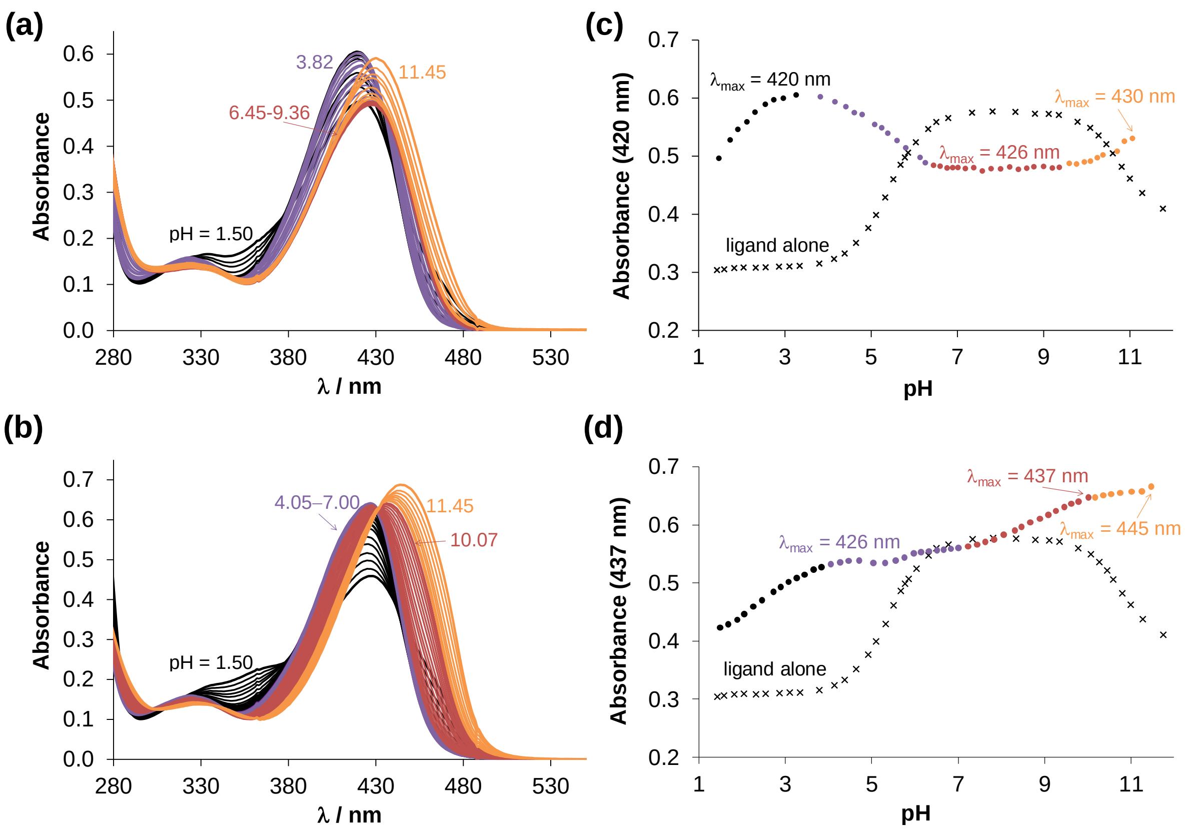

Figure S12. UV-vis spectra recorded for the Cu(II) – HQNO$_2$-L-Pro system in the pH range 1.5 – 11.5 at (a) 1:1 and (b) 1:2 Cu(II):ligand ratio. The corresponding absorbance values at (c) 420 nm (1:1) and (d) 460 nm (1:2) plotted against the pH are also shown in addition to the values of the free ligand (×) obtained from independent titrations. {c_{ligand} = 40 μM; $c_{\text{Cu(II)}}$ = 40 or 20 μM; I = 0.10 M KCl; l = 1 cm; t = 25.0 °C}

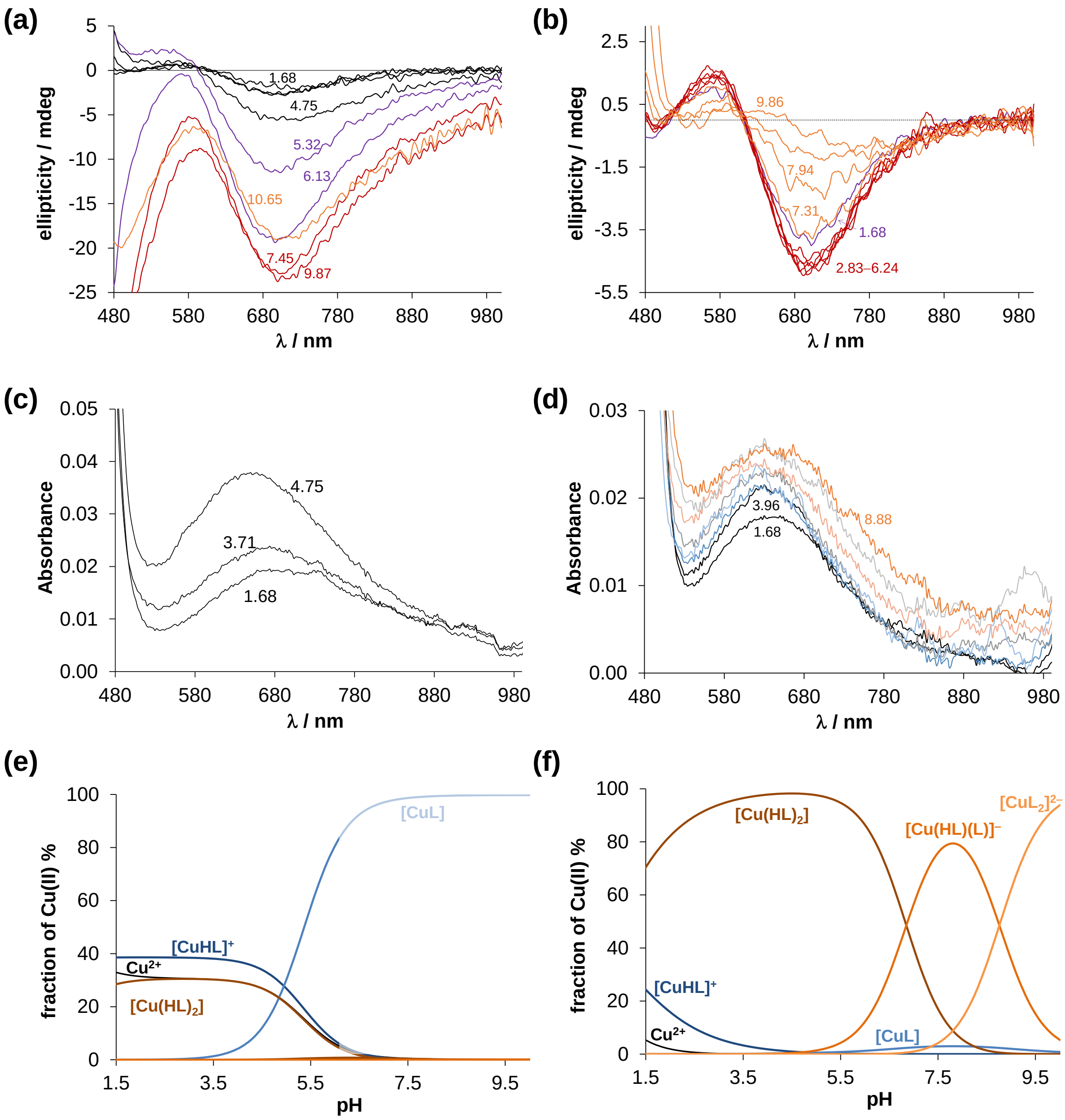

Figure S13. CD spectra recorded for the Cu(II) – HQNO2-L-Pro system in the pH range 1.6 – 10 at (a) 1:1 and (b) 1:2 Cu(II):ligand ratio in 30% (v/v) DMSO/H_2O. Visible absorption spectra measured for the same system at (c) 1:1 and (d) 1:2 Cu(II):ligand ratio and the corresponding concentration distribution curves at (e) 1:1 and (f) 1:2 Cu(II):ligand ratio obtained in water. The fainter part in (e) denotes the pH range where precipitate is formed. {c_{ligand} = 500 μM; $c_{Cu(II)}$ = 500 or 250 μM; I = 0.10 M KCl; l = 1 cm; t = 25.0 °C}

Table S3. Anisotropic EPR parameters of the complexes determined by the simulation of frozen solution EPR spectra recorded in Cu(II) – HQNO2-L-Pro system equilibrium system.[a] The coupling values are in 10^{-4} cm^{-1} unit.

	$g_{x,y}$	g_z	$A_{x,y}$/G	A_z/G	χ(°)	β(°)	*D*(G)	$d_{Cu\text{-}Cu}$(Å)	$g_{o,calc}$ [b]
$[Cu(aqua)]^{2+}$	2.081	2.424	13	110,6					2.195
monomer I	2.065	2.323	15	148					2.151
monomer II	2.060	2.245	20	175					2.151
dinuclear III	2.069	2.182	40	170	37	–13	75	6.5	2.129
	2.138		9						
dinuclear I	2.055	2.245	20	160	0	0	280	4.1	2.118
dinuclear II [c]	2.060	2.245	20	175	13	–16	267	4.3	2.122

[a] The experimental error were ±0.002 for g_x and g_y and ±0.001 for g_z, ± 2 G for A_x and A_y and ±1 G for A_z. [b] Calculated by the equation $g_{0,calc} = (g_x+g_y+g_z)/3$. [c] *g*- and *A*-values of monomer II was used for the simulation.

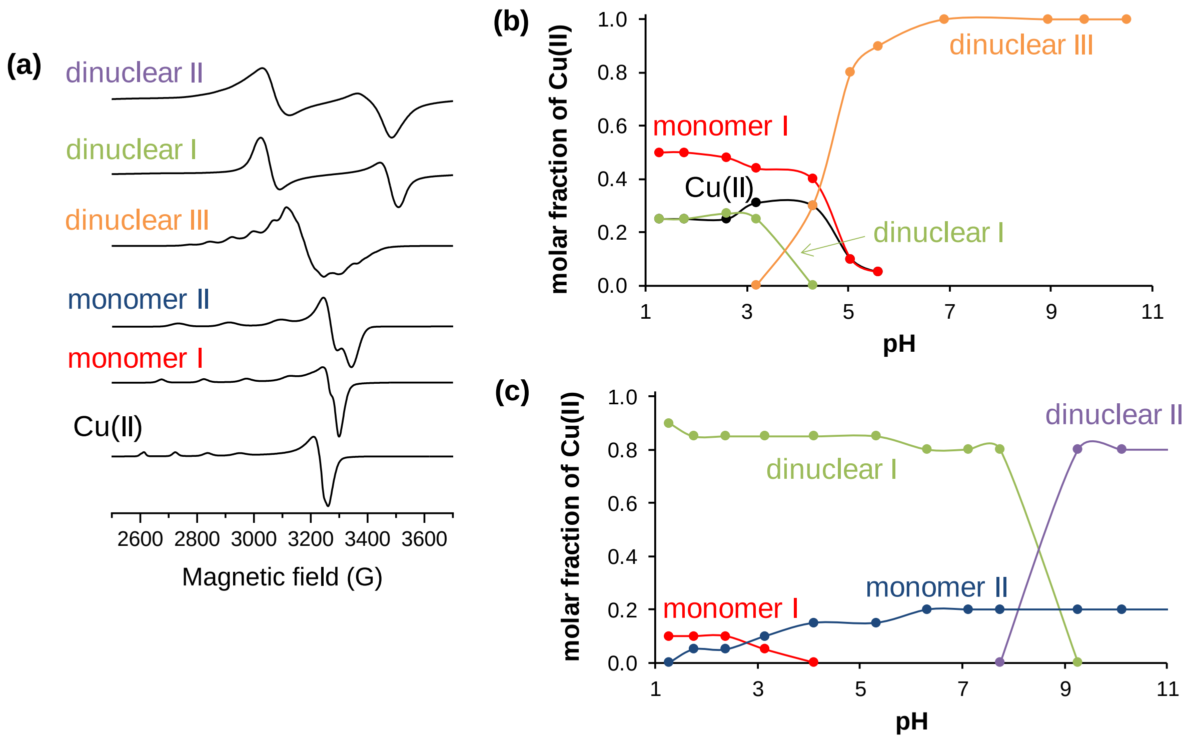

Figure S14. (a) Component anisotropic EPR spectra obtained from the simulation of frozen solution spectra in the Cu(II) – HQNO2-L-Pro system. Estimated distribution of Cu(II) species based on the EPR spectra at (b) 1:1 and (c) 1:2 Cu(II):ligand ratio.

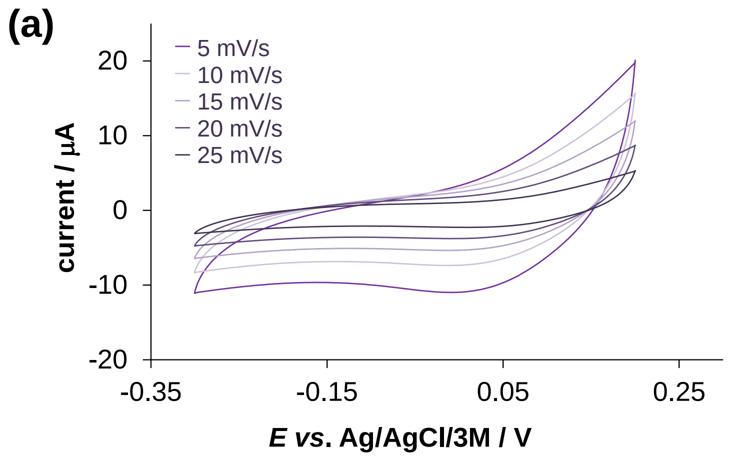

(b)

scan rate (V/s)	E_a (V)
**0.005**	0.043
**0.010**	0.018
**0.015**	0.023
**0.020**	0.025
**0.025**	0.006

Figure S15. (a) Cyclic voltammograms of the Cu(II) – HQNO2-L-Pro (1:2) systems at various scan rates (indicated in the figure). (b) The corresponding electrochemical data. {c_L = 1 mM, $c_{Cu(II)}$ = 0.5 mM; pH = 7.40 (10 mM HEPES); t = 25 °C; I = 0.1 M (TBAN)}.

Estimation of the competition between HQNO$_2$-L-Pro with human serum albumin (HSA) and transferrin (Tf) for Cu(II), Zn(II) and Fe(III)

The competition for the essential metal ions between serum proteins and the HQNO$_2$-L-Pro in the blood depends on their metal binding ability and the actual concentrations.

The total copper concentration in the blood is 10-22 μM, and is mostly bound to proteins (free serum copper: 1-2 μM), mainly to HSA, which is the most abundant protein in the serum (~630 μM). Through its so-called ATCUN motif, HSA binds Cu(II) with high affinity. The conditional constant log*K'* = 12 (pH 7.4, 100 mM NaCl) was reported by Bal et al. [67], and it is mentioned that 10-15% of plasma copper is bound to this protein. On the other hand, ceruloplasmin carries more than 40-70% of the total copper in healthy human plasma, however, its serum concentration is much lower (1–3 μM) compared to that of HSA [68]. This protein binds six Cu(II) ions, and the metal is bound by ceruloplasmin is considered as 'not exchangeable', which seriously complicates determination of the binding constant. Alpha-2-macroglobulin (1.3 μM) and low molecular components (e.g. histidine, 60-100 μM) also bind this metal ion. Thus, providing proper model calculation for the distribution of Cu(II) in the blood in the presence of the studied HQ-NO$_2$-L-Pro ligand is a rather difficult task. As we have found binding constant only for the Cu(II)-HSA adduct, the copper distribution was calculated for the Cu(II) – HQ-NO$_2$-L-Pro – HSA system using the stability constants of the Cu(II) – HQ-NO$_2$-L-Pro complexes determined in this work. The calculations were done at 2 μM and 22 μM Cu(II) and 630 μM HSA concentrations, respectively, varying the concentration of HQ-NO$_2$-L-Pro (Figure S16). *This estimation shows that the title ligand would be able to compete with HSA for Cu(II).*

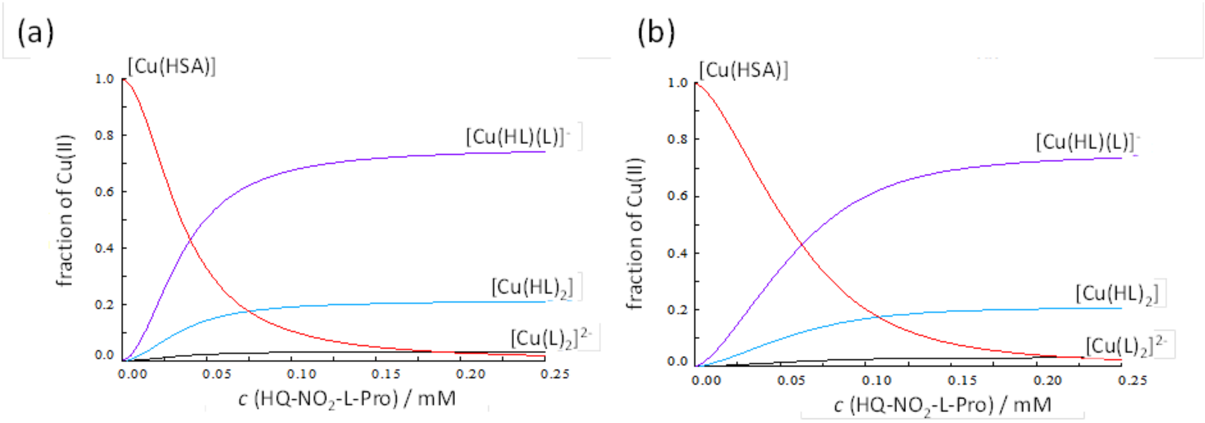

Figure S16. Estimated concentration distribution curves for the Cu(II) – HQ-NO$_2$-L-Pro – HSA system at (a) 2 μM and (b) 22 μM concentration of Cu(II), 630 μM HSA at pH 7.4 by varying the concentration of HQ-NO$_2$-L-Pro (0–250 μM).

The Zn(II) serum concentration (~12-25 μM) falls into the similar range as that of Cu(II) and this metal ion is bound to both low (e.g. histidine, cysteine) and high molecular mass components (e.g. HSA, transferrin, α-macroglobulin) of the blood [69]. The Zn(II) binding constants for HSA and apotransferrin are available in the literature (apoTf: log*K'*1: 7.8, log*K'*2: 6.4 (0.015 M HCO_3^-; pH 7.4 [70]; HSA log*K'*: 7.1-7.9 [71, 72]) and based on these data the Zn(II) distribution was estimated for the Zn(II) – HQ-NO$_2$-L-Pro – HSA – transferrin system at 630 μM HSA and 37 μM transferrin concentration, respectively, assuming that 1/3 of the binding site of the latter protein is occupied by Fe(III) (Figure S17.a). *It shows that the title ligand would able to compete with HSA and/or transferrin at higher concentrations for the Zn(II).*

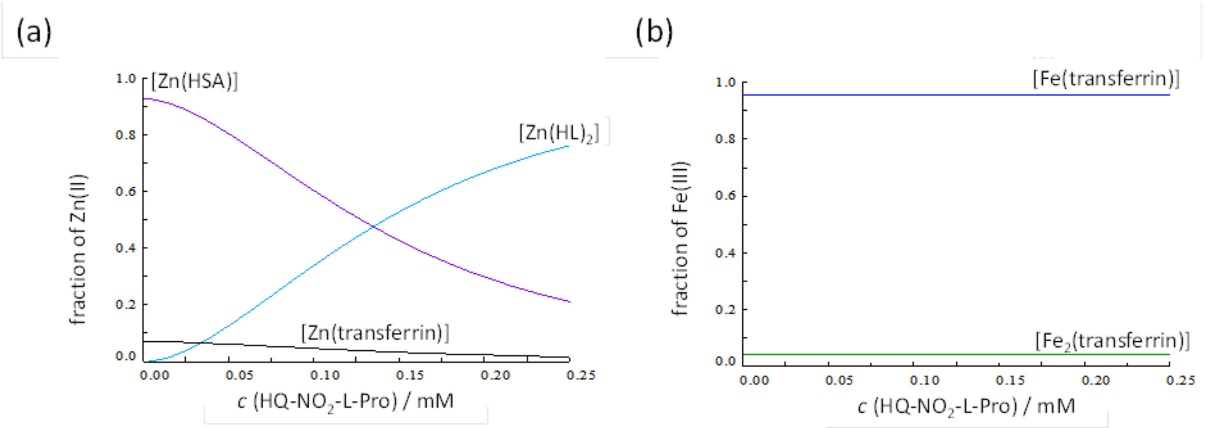

Figure S17. Estimated concentration distribution curves for the (a) Zn(II) – HQ-NO2-L-Pro – HSA – transferrin system at c_{Zn} = 25 µM, c_{HSA} = 630 µM, c_{Tf} = 37 µM concentrations and (b) for the Fe(III) – HQ-NO2-L-Pro – transferrin system at c_{Fe} = 12 µM, c_{Tf} = 37 µM concentrations by varying the concentration of HQ-NO2-L-Pro (0–250 µM).

In the blood the iron concentration is usually in the range of 10-30 µM and mostly bound to transferrin as Fe(III) at two binding sites with very high efficacy (apoTf: log*K*'1: 20.7, log*K*'2: 19.4 (0.015 M HCO_3^-; pH 7.4 [73]). Assuming that 2/3 of the metal binding sites of Tf is unoccupied by Fe(III), we can also estimate the distribution of Fe(III) between this protein and the title ligand (Figure S17.b). This estimation suggests that the ligand is not able to replace Fe(III) from the binding sites of Tf as it was expected.

With these model calculations we can point out that the title ligand might interact with Zn(II) and Cu(II) and not with Fe(III) among the mentioned essential metal ions in the blood serum. However, we would caution the reader to draw any serious conclusions from these predictions, since for the adequate prediction all possible interactions should be considered including complex formation with both the low and high molecular mass components of the serum, and in many cases the required equilibrium constants are not available.

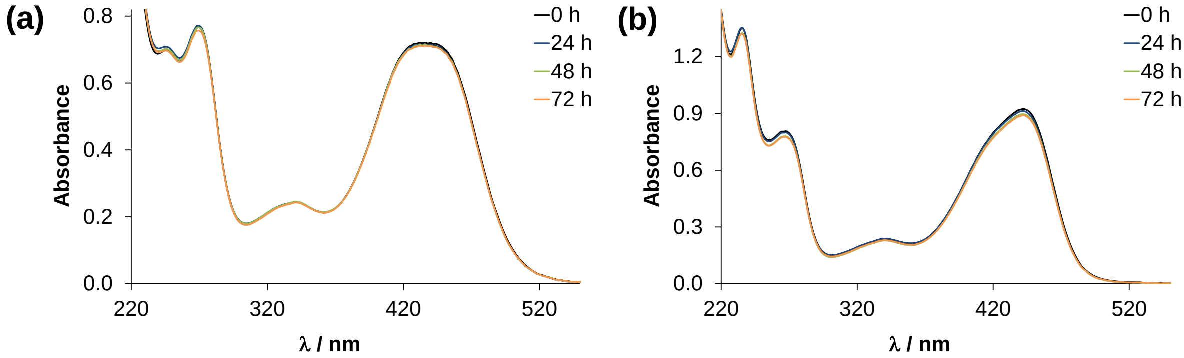

Figure S18. UV-vis spectra of the (a) RuCym and (b) RhCp* complex of HQNO2-L-Pro recorded in time (indicated in the figure) at pH 7.4. {$c_{complex}$ = 50 µM; PBS' buffer; l = 1 cm; t = 25.0 °C}

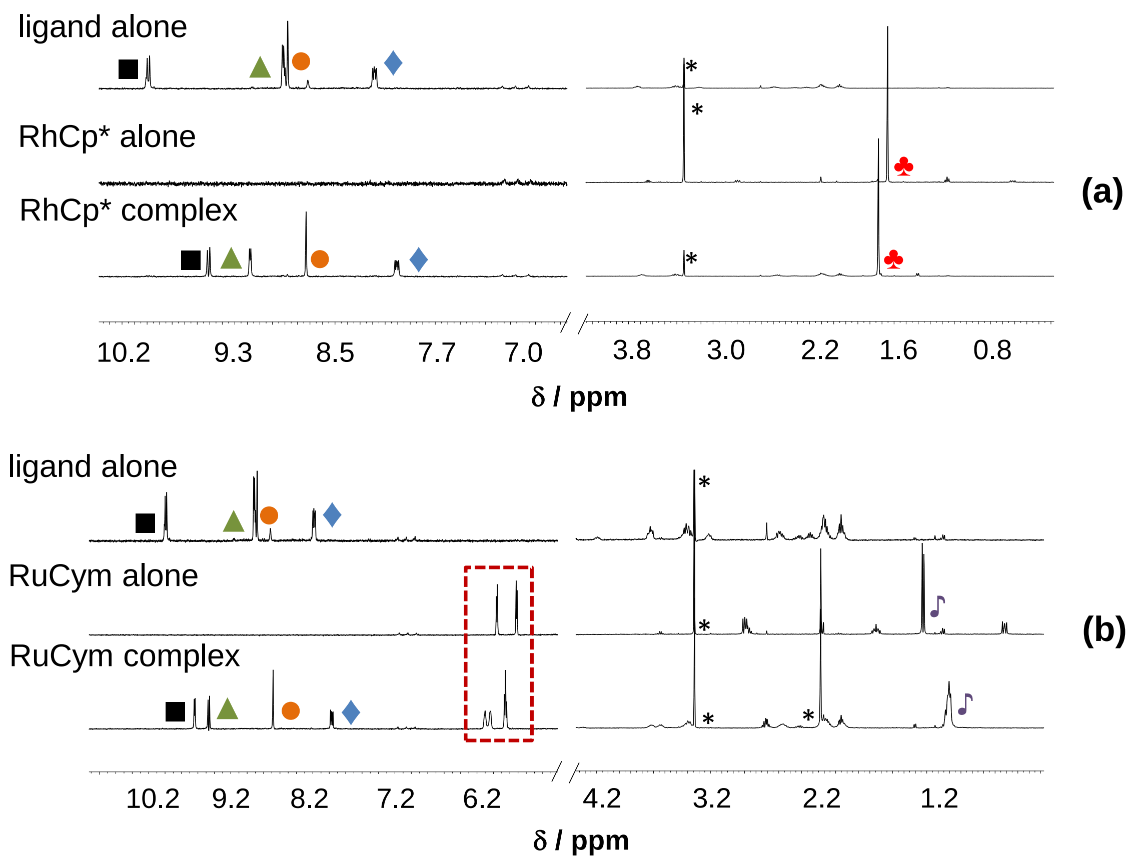

Figure S19. ^{1}H NMR spectra recorded for the (a) RhCp* and (b) RuCym complex of HQNO2-L-Pro at pH 1 in 10% (v/v) D_2O/H_2O. Peak assignation for the ligand (free or complexed) shown in Figure S9.b, the framed peaks belong to the *p*-cymene aromatic CH protons, and the methyl protons in the RhCp* (♣) and RuCym (♪) cores are also shown in the up-field part of the spectra. * Denotes solvent peaks. {$c_{\text{complex or ligand}}$ = 500 μM; I = 0.20 M KNO_3; t = 25.0 °C}

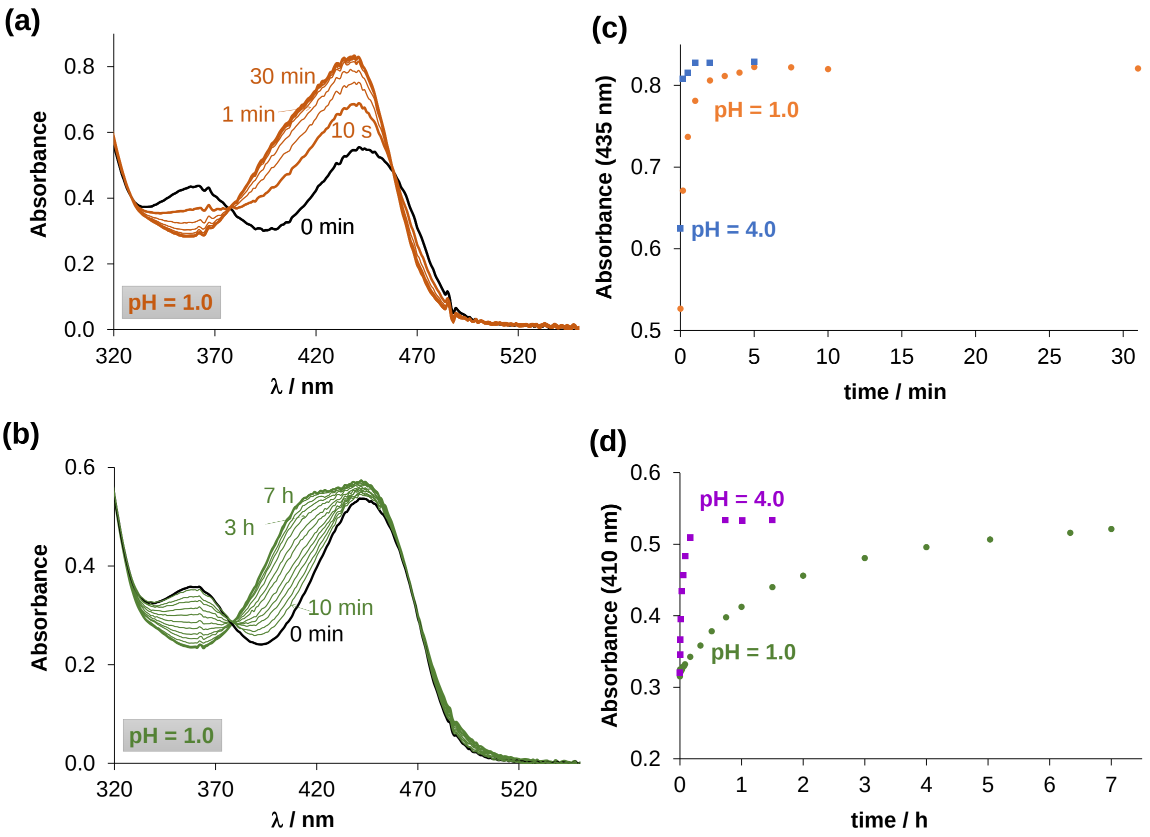

Figure S20. UV-vis spectra recorded for the (a) RhCp* – $HQNO_2$-L-Pro (1:1) and (b) RuCym – $HQNO_2$-L-Pro (1:1) systems at different times at pH 1. Absorbance values plotted against the time measured in the (c) RhCp* – $HQNO_2$-L-Pro (1:1) at 435 nm, and (d) RuCym – $HQNO_2$-L-Pro (1:1) at 410 nm at pH 1 and 4 (indicated in the figure). {c_{ligand} = 50 µM; $c_{\text{RhCp* or RuCym}}$ = 50 µM; l = 1 cm; t = 25.0 °C}

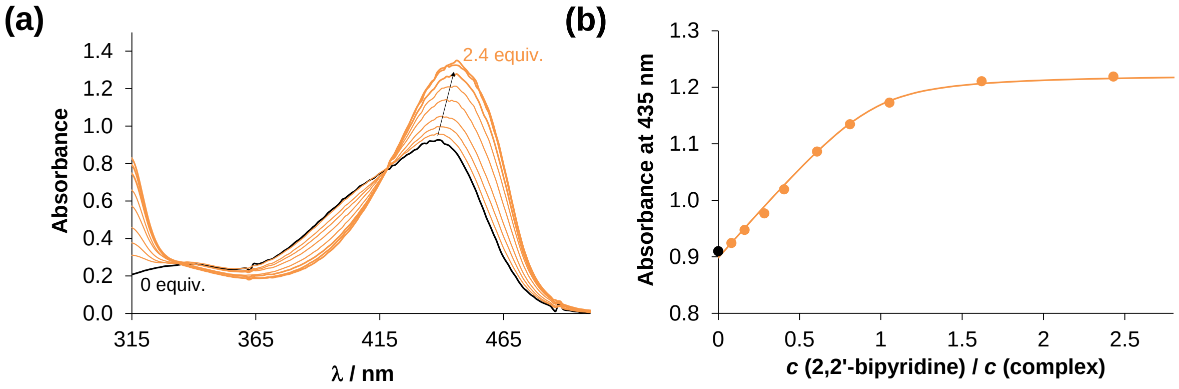

Figure S21. (a) UV-vis spectra of the RhCp*-$HQNO_2$-L-Pro complex at various equivalents of 2,2′-bipyridine at pH 7.4 after 24 h waiting time and (b) the measured absorbance values at 435 nm plotted against the 2,2′-bipyridine:complex ratio. LogK' = 11.89±0.02 was calculated for the RhCp*-$HQNO_2$-L-Pro complex based on the spectral changes using the stability constants of the RhCp*-2,2′-bipyridine complex taken from Ref. [74]. {c_{complex} = 50 µM; PBS′ buffer; l = 1 cm; t = 25.0 °C}

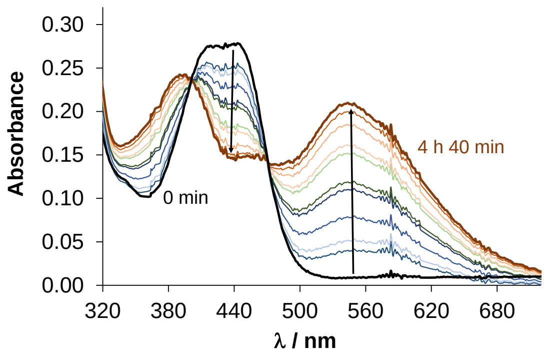

Figure S22. Time-dependent UV-vis spectra of the RuCym-HQNO2-L-Pro complex on the presence of two equivalents of 2,2′-bipyridine at pH 7.4 measured in a tandem cuvette. The development of the band at 550 nm is typical for the process when the arene ring is replaced by the competitor ligand and the formation of a mixed-ligand Ru-HQNO2-L-Pro-2,2′bipyridine complex. {$c_{complex}$ = 25 μM; $c_{2,2'\text{-bipyridine}}$ = 50 μM; PBS′ buffer; l = 1 cm; t = 25.0 °C}

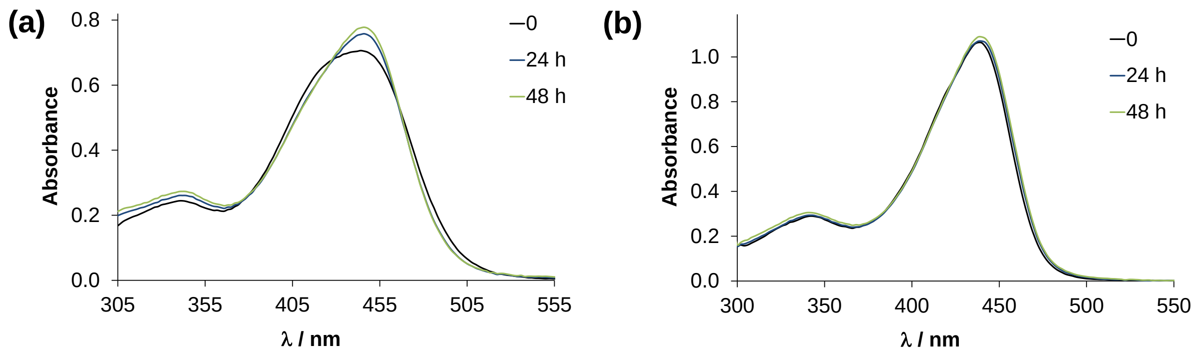

Figure S23. UV-vis spectra of the (a) RuCym and (b) RhCp* complex of HQNO2-L-Pro recorded in time (indicated in the figure) in blood serum (four-time diluted with PBS′ buffer). (Background corrected spectra.) {c = 40 μM; l = 1 cm; t = 25.0 °C}

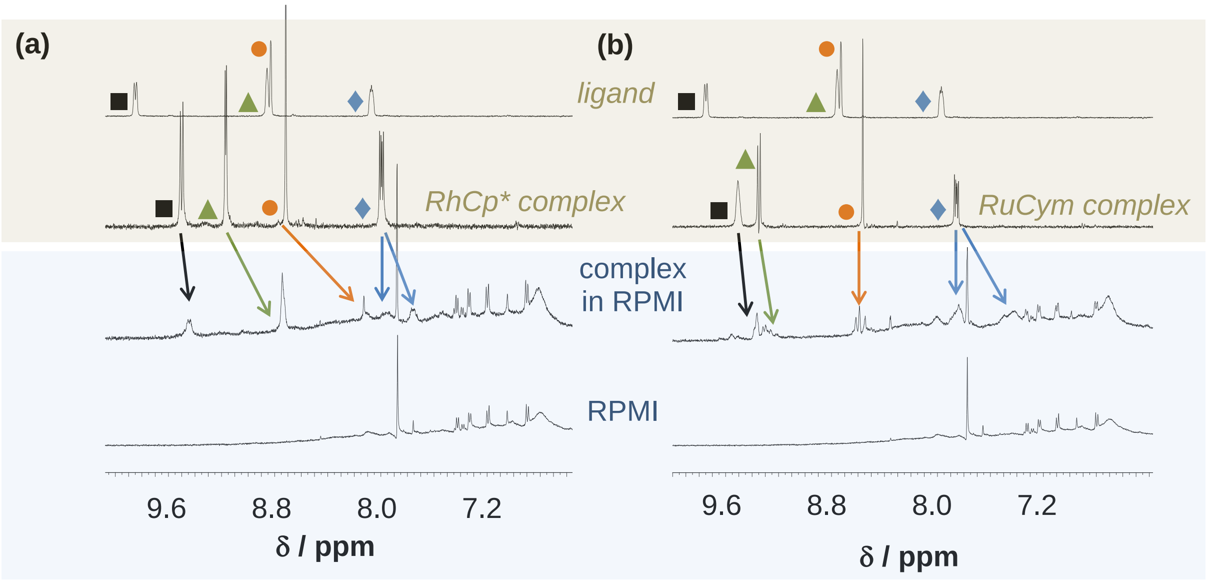

Figure S24. High-field region of the ^{1}H NMR spectra recorded for the (a) RhCp* and (b) RuCym complex of HQNO2-L-Pro in RPMI medium after 24 h incubation time. Peak assignation for the ligand (free or complexed) shown in Figure S9.b. Spectra of RPMI, the ligand (pH 7.4) and complex (pH 7.4) are also shown for comparison. {$c_{\text{complex or ligand}}$ = 500 μM; 10% (v/v) D_2O/H_2O; t = 25.0 °C}

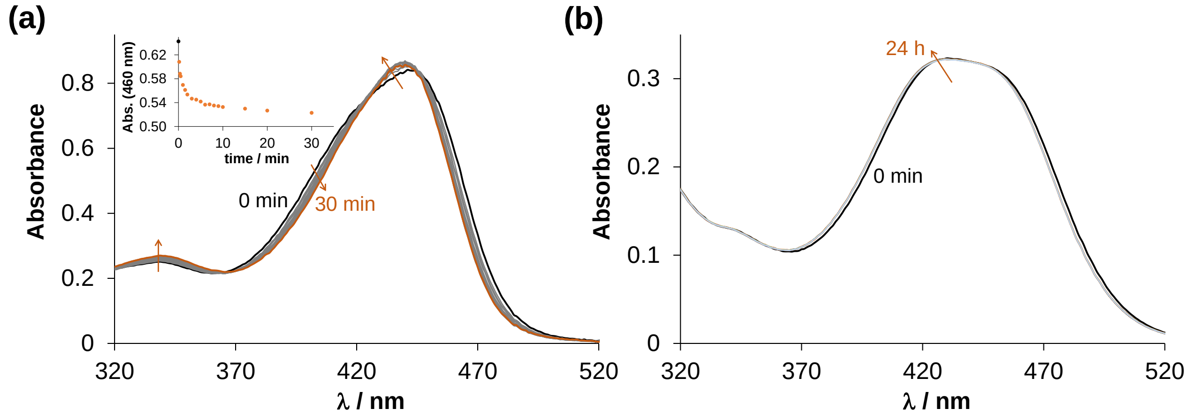

Figure S25. UV-vis spectra recorded for the (a) RhCp* complex and the (b) RuCym complex of HQNO2-L-Pro upon addition of HSA at pH 7.4. Inserted figure shows the absorbance values at 460 nm plotted against the time. {(a) c_{complex} = 50 μM; c_{HSA} = 25 μM; (b) c_{complex} = 25 μM; c_{HSA} = 25 μM; PBS′ buffer; l = 1 cm; t = 25.0 °C}

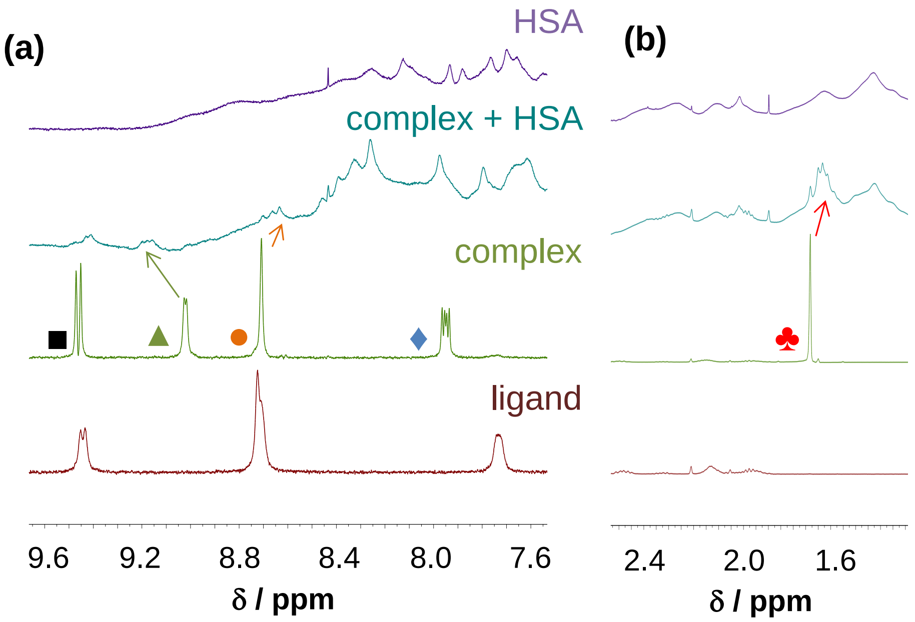

Figure S26. (a) High-field and (b) low-field region of the ^{1}H NMR spectra recorded for the RhCp* complex of HQNO2-L-Pro in the presence of HSA after 24 h incubation time. Peak assignation for the ligand (free or complexed) shown in Figure S9.b. Spectra of HSA, the ligand (pH 7.4) and complex (pH 7.4) are also shown for comparison. Comparing the spectra neither free ligand nor metal precursor is seen in the complex–HSA system, however, the peaks belonging to the original complex are shifted indicating the formation of mixed-ligand species. {$c_{\text{complex or ligand}}$ = 1000 μM; c_{HSA} = 500 μM; PBS' buffer; 10% (v/v) D_2O/H_2O; t = 25.0 °C}

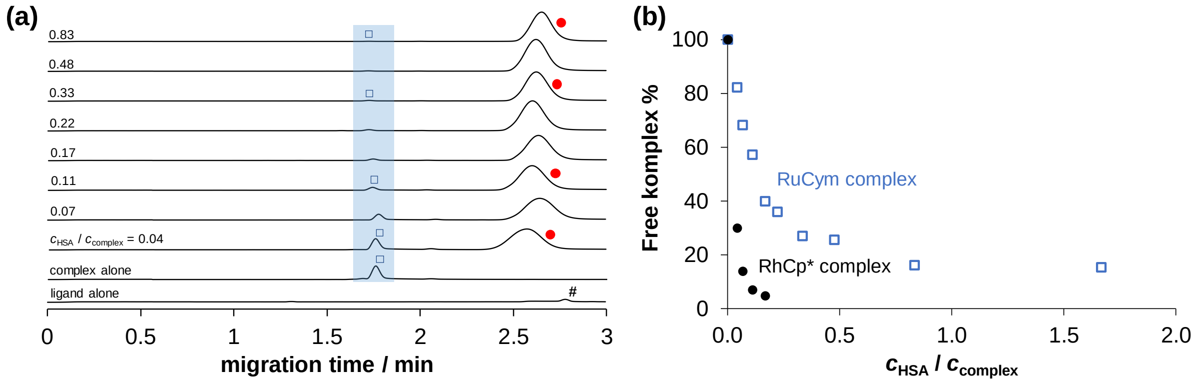

Figure S27. (a) Electropherograms of free ligand, RuCym complex of HQNO2-L-Pro and the complex in the presence of HSA at various complex-to-HSA ratios after 24 h incubation time. Symbols indicate the free complex (□), free ligand (#) and HSA & HSA-complex adduct (●). Peaks belonging to the free complex were assigned based on their UV-vis spectra measured at the peak maxima. (b) Fraction of the free (unbound) complex in the same system (□) and for the RhCp* complex under the same condition for comparison (●). {c_{HSA} = 30 μM; c_{complex} = 18 – 720 μM; pH = 7.40 (20 mM phosphate buffer); λ = 200 nm; t = 25.0 °C}

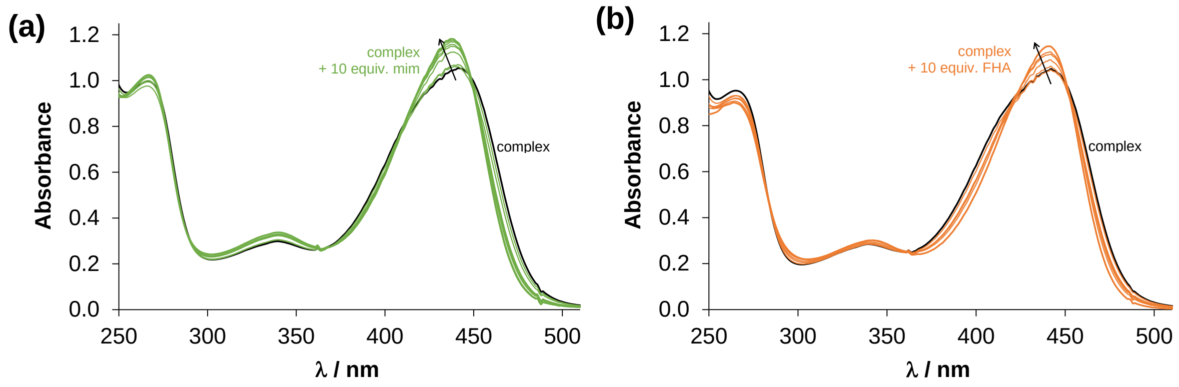

Figure S28. UV-vis spectra recorded for the RhCp* complex of HQNO$_2$-L-Pro upon addition of (a) mim and (b) FHA at pH 7.4. Based on the observed spectral changes conditional stability constants were computed for the [RhCp*(L)(H$_2$O/Cl)]$^+$ + co-ligand ⇌ [RhCp*(L)(co-ligand: mim or FHA)] + H$_2$O/Cl$^-$ equilibrium process. {$c_{complex}$ = 55 µM; $c_{mim\ or\ FHA}$ = 0 – 550 µM; PBS′ buffer; l = 1 cm; t = 25.0 °C}

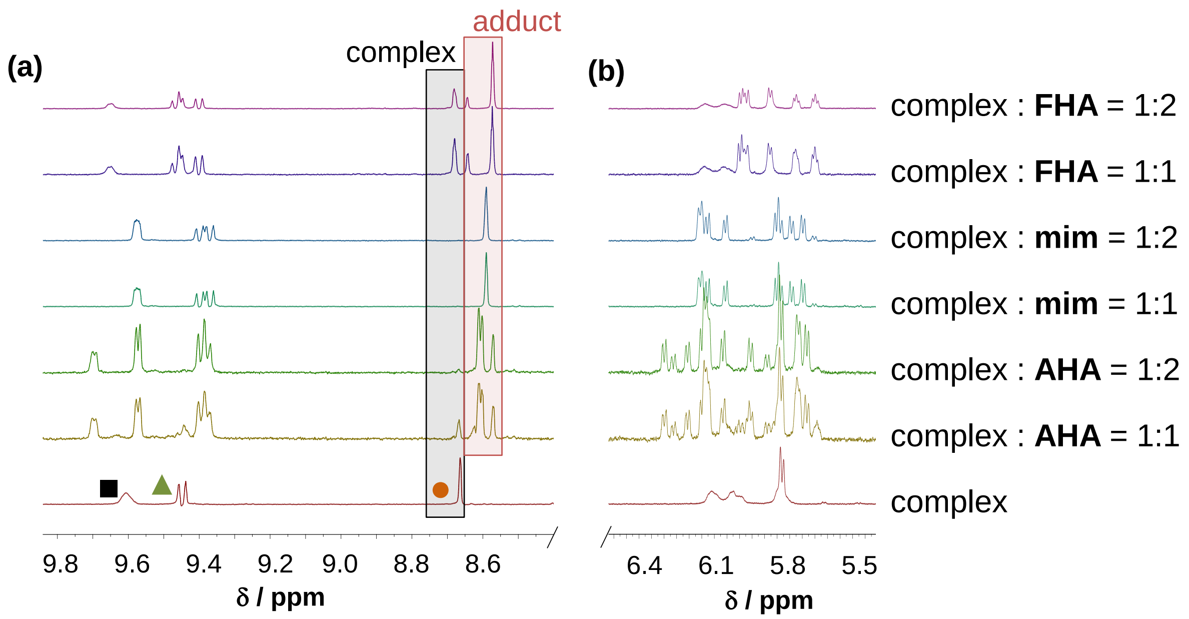

Figure S29. ^{1}H NMR spectra recorded for the RuCym complex of HQNO$_2$-L-Pro at pH 7.4 in 10% (v/v) D$_2$O/H$_2$O using 24 h incubation time in the presence of one or two equivalents of binding model: FHA, mim or AHA in the region of the (a) CH(2) (■), CH(4) (▲) and CH(6) (●) and (b) *p*-cymene ring CH protons. In the selected regions the binding models have no peaks. The framed peaks belong to the protons of the complex alone (black) and the mixed-ligand adduct formed with the binding model (red). {$c_{complex}$ = 0.5 mM; $c_{binding\ model}$ = 0.5 or 1.0 mM; PBS′ buffer; t = 25.0 °C}

Table S4. Formation constants (log*β*) of the various hydroxido species of Fe(III), Fe(II) and RuCym and RhCp*. {t = 25.0 °C}

**log*β***	**Fe(III)** [a]	**Fe(II)** [b]	**RuCym** [c]	**RhCp*** [d]
MH_{-1}	–3.22	–9.43	–	–
MH_{-2}	–6.73	–20.73	–	–
MH_{-3}	–	–32.68	–	–
M_2H_{-2}	–4.09	–	–	–8.53
M_2H_{-3}	–	–	–9.16	–14.26
M_3H_{-4}	–7.58	–	–	–

[a] Data taken from [56], I = 0.2 M KCl. [b] Data taken from [57], I = 0 M. [c] Data taken from [58], I = 0.2 M KNO_3. [d] Data taken from [59], I = 0.2 M KNO_3.

References

[12] Mészáros, J.P.; Poljarevic, J.M.; Szatmári, I.; Csuvik, O.; Fülöp, F.; Szoboszlai, N.; Spengler, G.; Enyedy, É.A. An 8-hydroxyquinoline–proline hybrid with multidrug resistance reversal activity and the solution chemistry of its half-sandwich organometallic Ru and Rh complexes. *Dalton Trans*. **2020**, *49*, 7977–7992.

[56] Baes, C.F.; Mesmer, R.E. *The Hydrolysis of Cations*. Wiley, New York, 1976.

[57] Brown, P.L.; Ekberg, C. *Hydrolysis of Metal Ions*. Wiley, **2016**, 573–585.

[58] Bíró, L.; Godó, A.J.; Bihari, Z.; Garribba, E.; Buglyó, P. Tuning the hydrolytic properties of half-sandwich-type organometallic cations in aqueous solution. *Eur. J. Inorg. Chem*. **2013**, *2013*, 3090–3100.

[59] Dömötör, O.; Aicher, S.; Schmidlehner, M.; Novak, M.S; Roller, A.; Jakupec, M.A.; Kandioller, W.; Hartinger, C.G.; Keppler, B.K.; Enyedy, É.A. Antitumor pentamethylcyclopentadienyl rhodium complexes of maltol and allomaltol: synthesis, solution speciation and bioactivity. *J. Inorg. Biochem*. **2014**, *134*, 57–65.

[67] Rózga, M.; Sokołowska, M.; Protas, A.M.; Bal, W. Human serum albumin coordinates Cu(II) at its N-terminal binding site with 1 pM affinity. *Biol. Inorg. Chem*. **2007**, *12*, 913–918.

[68] Linder, M.C. Ceruloplasmin and other copper binding components of blood plasma and their functions: an update. *Metallomics* **2016**, *8*, 887–905.

[69] Boyett; J.D.; Sullivan, J.F. Distribution of protein-bound zinc in normal and cirrhotic serum. *Metabolism* **1970**, *19*, 148–157.

[70] Harris, W.R. Thermodynamic binding constants of the zinc-human serum transferrin complex. *Biochemistry* **1983**, *22*, 3920–3926.

[71] Masuoka, J.; Saltman, P. J. Zinc(II) and copper(II) binding to serum albumin. A comparative study of dog, bovine, and human albumin. *J. Biol. Chem.* **1994**, *269*, 25557–25561.

[72] Ohyoshi, E.; Hamada, Y.; Nakata, K.; Kohata, S. The interaction between human and bovine serum albumin and zinc studied by a competitive spectrophotometry. *J. Inorg. Chem*. **1999**, *75*, 213–218.

[73] Aisen, P.; Leibman, A.; Zweier, J. Stoichiometric and site characteristics of the binding of iron to human transferrin. *J. Biol. Chem*. **1978**, *253*, 1930–1937.

[74] Enyedy, É.A.; Mészáros, J. P.; Dömötör, O.; Hackl, C.M., Roller, A.; Keppler, B.K.; Kandioller, W. Comparative solution equilibrium studies on pentamethylcyclopentadienyl rhodium complexes of 2, 2'-bipyridine and ethylenediamine and their interaction with human serum albumin. *J. Inorg. Biochem.* **2015**, *152*, 93-103.
